# A Stable Open-Shell Conjugated Diradical Polymer with Ultra-High Photothermal Conversion Efficiency for NIR-II Photo-Immunotherapy of Metastatic Tumor

**DOI:** 10.1007/s40820-023-01219-x

**Published:** 2023-11-20

**Authors:** Yijian Gao, Ying Liu, Xiliang Li, Hui Wang, Yuliang Yang, Yu Luo, Yingpeng Wan, Chun-sing Lee, Shengliang Li, Xiao-Hong Zhang

**Affiliations:** 1 College of Pharmaceutical Sciences, Soochow University, Suzhou, 215123 People’s Republic of China; 2 Institute of Functional Nano & Soft Materials (FUNSOM), Soochow University, Suzhou, 215123 People’s Republic of China; 3 Center of Super-Diamond and Advanced Films (COSDAF) & Department of Chemistry, City University of Hong Kong, 83 Tat Chee Avenue, Kowloon, Hong Kong SAR, People’s Republic of China

**Keywords:** NIR-II conjugated polymer, Photothermal, Radical, Nanoparticles, Cancer therapy

## Abstract

Massive efforts have been concentrated on the advance of eminent near-infrared (NIR) photothermal materials (PTMs) in the NIR-II window (1000–1700 nm), especially organic PTMs because of their intrinsic biological safety compared with inorganic PTMs. However, so far, only a few NIR-II-responsive organic PTMs was explored, and their photothermal conversion efficiencies (PCEs) still remain relatively low. Herein, donor–acceptor conjugated diradical polymers with open-shell characteristics are explored for synergistically photothermal immunotherapy of metastatic tumors in the NIR-II window. By employing side-chain regulation, the conjugated diradical polymer TTB-2 with obvious NIR-II absorption was developed, and its nanoparticles realize a record-breaking PCE of 87.7% upon NIR-II light illustration. In vitro and in vivo experiments demonstrate that TTB-2 nanoparticles show good tumor photoablation with navigation of photoacoustic imaging in the NIR-II window, without any side-effect. Moreover, by combining with PD-1 antibody, the pulmonary metastasis of breast cancer is high-effectively prevented by the efficient photo-immunity effect. Thus, this study explores superior PTMs for cancer metastasis theranostics in the NIR-II window, offering a new horizon in developing radical-characteristic NIR-II photothermal materials.

**Supplementary Information:**

The online version contains supplementary material available at 10.1007/s40820-023-01219-x.

## Highlights

- By employing side-chain regulation, the photothermal therapy response of conjugated diradical polymer can red-shift from near-infrared (NIR)-I to the NIR-II region. This strategy offers new radical materials in developing NIR-II photothermal materials.
- The conjugated diradical polymer TTB-2 receives a record-high photothermal conversion efficiency of 87.7%, far beyond the recently reported NIR-II photothermal agents. Such conjugated diradical polymer must enrich the NIR-II photothermal agents’ library and provide important insight for more diradical polymer construction.
- The conjugated diradical polymer nanoparticles TTB-2 NPs achieve good tumor photoablation with navigation of photoacoustic imaging in the NIR-II window, without any side effect. Furthermore, the efficient photo-immunity effect prevents the pulmonary metastasis of breast cancer.

**Supplementary Information:**

The online version contains supplementary material available at 10.1007/s40820-023-01219-x.

## Graphical abstract

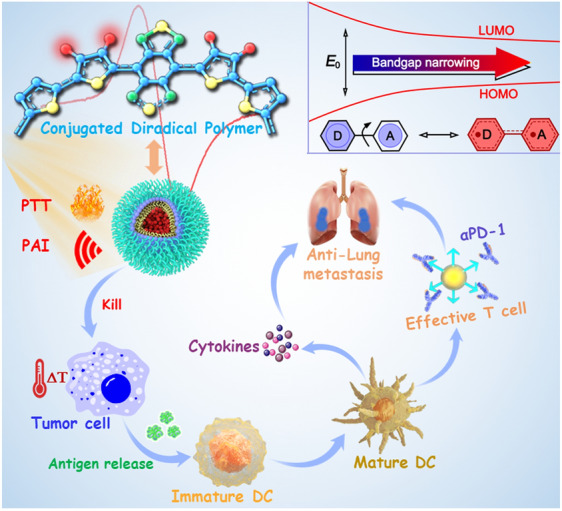

## Introduction

Photothermal therapy (PTT) providing non-invasive and temporal-spatial resolution characteristics for cancer treatments, is widely regarded as a promising therapeutic due to its unique advantages beyond traditional treatments [1–11]. Great advances in PTT have been made in recent years, in which various PTT agents mainly including metal nanoparticles [12–14], transition metal dichalcogenides (TMDCs) [15–18], carbon materials and their analogs [19–22], Mxenes [23, 24], and conjugated molecular materials [25–32], have been extensively explored for PTT applications. Among these PTT agents, organic PTT agents (PTAs), such as near-infrared (NIR) dye, conjugated polymers, and small molecular materials, have raised excessive concerns because of their inherent biocompatibility and flexible preparation [33–40]. To achieve high PTT performance, several strategies in materials design and nanoparticle preparation of organic PTT agents including conjugation extending, molecular motor appending, radiative transition inhibiting, and aggregate stacking, were closely explored [4, 7, 38–41]. However, the photothermal conversion efficiencies (PCEs) of current organic PTT agents considerably remain relatively low level, especially with good responses in NIR-II (1000–1700 nm) window which provides much higher penetration and bigger permissible light dose compared to NIR-I [42–44]. Moreover, exploring new PTT mechanisms for boosting PCEs is still tremendously missing. Therefore, exploring new organic PTT agents with high NIR-II performance is still in urgent need.

Organic radicals, characterized by open-shell and radical characteristics, have already been widely applied in organic light-emitting diodes (OLEDs) [45–47], organic electronics [48, 49], batteries spintronics [50], and quantum devices [51–53] because of their unique physicochemical properties. Recent advances have demonstrated that radical-characterized organic materials generally hold notably narrower bandgaps than that of organic materials with normally closed shell [54–59], and endowing organic radicals with great promise in abundant NIR applications. Zhang’s group [60] reported a stable-enhanced organic radical via supramolecular host–guest interaction to develop NIR photothermal materials. And Yin’s group [61] prepared a crystalline metal–organic frameworks (MOFs) material integrated with radical anions for photothermal therapy application. Through molecular design, stable radical cation-containing covalent organic frameworks (COFs) [62] and organic diradicals [63–65] have also been explored for efficient NIR photothermal applications. However, the reported studies mainly concentrate on the NIR-I region (700–1000 nm), which limits their biomedical applications in the deep-tissue. Moreover, a new strategy to prolong the stability of organic radical has been rarely reported. Thus, exploration of stable organic radicals with good response in the NIR-II window is highly desirable but still challenging.

**Scheme 1:**
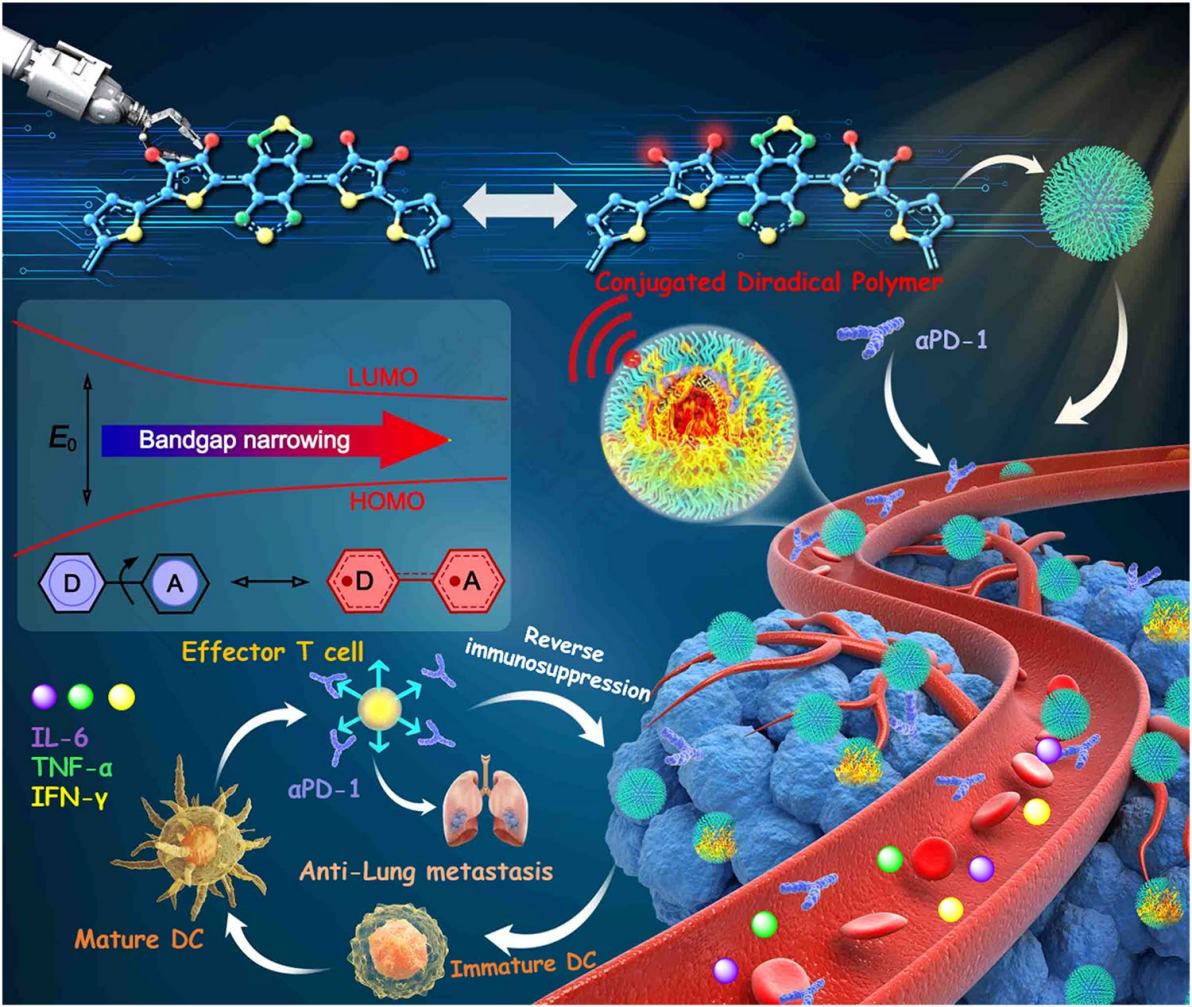
Schematic showing stable conjugated diradical polymers for NIR-II photo-immunotherapy of metastatic tumor

To overcome these shortcomings, we successfully develop a conjugated diradical polymer with 87.7% photothermal conversion efficiency for efficient photothermal immunotherapy of metastatic tumors in the NIR-II window (Scheme 1). We engineer D–A conjugated diradical polymers (TTB-1, and TTB-2) by side-chain regulation to red-shift the PTT response from NIR-I to the NIR-II region. After assembling the conjugated d diradical polymers into water-soluble nanoparticles (NPs), the TTB-2 NPs achieve superior NIR-II photothermal conversion efficiency of 87.7% with good stability. In vitro and in vivo experiments demonstrate that the TTB-2 NPs realize high-performance tumor photoablation and photoacoustic (PA) imaging in the NIR-II window, without any side-effect. Moreover, by combining NIR-II PTT of TTB-2 NPs with PD-1, the pulmonary metastasis of breast cancer is highly effective and prevented via the photo-immunity effect. Thus, this study offers a new horizon for developing the first conjugated diradical polymer with efficient NIR-II activated photo-immunity theranostics of tumor metastasis.

## Experimental Section

### Instrumentations

UV–vis–NIR absorption was conducted on a PE 950 UV–vis–NIR spectroscope. The size of TTB NPs was determined by dynamic light scattering (DLS) on a Malvern ZetaSizer Nano ZS90 system. Morphological characterization of TTB-1 and TTB-2 NPs was performed by transmission electron microscopy (TEM). Fluke Ti480 pro thermal imager was used to obtain all thermal images.

### Synthesis of TTB-1

Under N_2_ atmosphere, 4,8-bis(5-bromo-3-(2-ethylhexyl)-2-thienyl)-2λ4δ2-Benzo[1,2-c:4,5-c']bis [1, 2, 5] thiadiazole (74 mg, 0.1 mmol) and 5,5'‐bis(triMethylstannyl)‐2,2'‐bithiophene (49 mg, 0.1 mmol) were dissolved in anhydrous toluene (10 mL) in a 25-mL necked flask. Continue to inject N_2_ for 10 min, Pd (PPh_3_)_4_ (12 mg, 0.01 mmol) was added slowly into the flask. Then, the mixture was stirred at 110 °C for 24 h. After that, the mixture was cooled to room temperature, washed with water and chloroform. Add anhydrous MgSO_4_ into CHCl_3_ to remove water and filter, then add 40 mL methanol to precipitate the crude product after concentration. Afterward, the product was collected and further purified by Soxhlet extraction with CH_3_OH, n-hexane, acetone and chloroform, following by recrystallization in acetonitrile to obtain a green solid. ^1^H NMR (400 MHz, CDCl_3_) δ 7.29 (s, 2H), 7.22 (s, 2H), 7.17 (s, 2H), 2.57 (s, 4H), 1.25 (s, 18H), 0.66 (s, 6H), 0.49 (s, 6H). GPC (THF, polystyrene standard), Mw: 1.02 × 10^4^ g mol^–1^, PDI: 1.08.

### Synthesis of TTB-2

Under N_2_ atmosphere, 4,8-bis (5-bromo-4- (2-octyldodecyl) thiophenyl) - benzo [1,2-c; 4,5-c '] di [1,2,5] thiadiazole (107 mg, 0.1 mmol) and 5,5'‐bis(triMethylstannyl)‐2,2'‐bithiophene (49 mg, 0.1 mmol) were dissolved in anhydrous toluene (10 mL) in a 25-mL necked flask. Continue to inject N_2_ for 10 min, Pd (PPh_3_)_4_ (12 mg, 0.01 mmol) was added slowly into the flask. Then, the mixture was stirred at 110 °C for 24 h. After that, the mixture was cooled to room temperature, washed with water and chloroform. Add anhydrous MgSO_4_ into CHCl_3_ to remove water and filter, then add 40 mL methanol to precipitate the crude product after concentration. Afterward, the product was collected and further purified by Soxhlet extraction with CH_3_OH, n-hexane, acetone and chloroform, following by recrystallization in acetonitrile to obtain a brown solid. ^1^H NMR (400 MHz, CDCl_3_) δ 7.72–7.69 (m, 2H), 7.44–7.36 (m, 4H), 2.76 (s, 4H), 1.89 (s, 2H), 1.25 (s, 64H), 0.85 (s, 12H). GPC (THF, polystyrene standard), Mw: 0.96 × 10^4^ g mol^–1^, PDI: 1.05.

### Preparation of TTB NPs

The TTB in THF (0.5 mg mL^−1^, 1 mL) and DSPE-mPEG_2000_ (5.0 mg mL^−1^, 1 mL) in THF were treated by ultrasonication for 10 min. Evenly mix the above solutions and ultrasonic for another 5 min and slowly added dropwise into ultrapure water (18 mL). After stirring at room temperature for 48 h, filter with 0.22 μm microporous membrane, and concentrate with centrifugal filter units (Millipore, size of 100 kDa). Finally, the concentrated TTB NPs dispersion is stored at 4 °C under darkness.

## Results and Discussion

### Design and Structural Characterizations

**Fig. 1:**
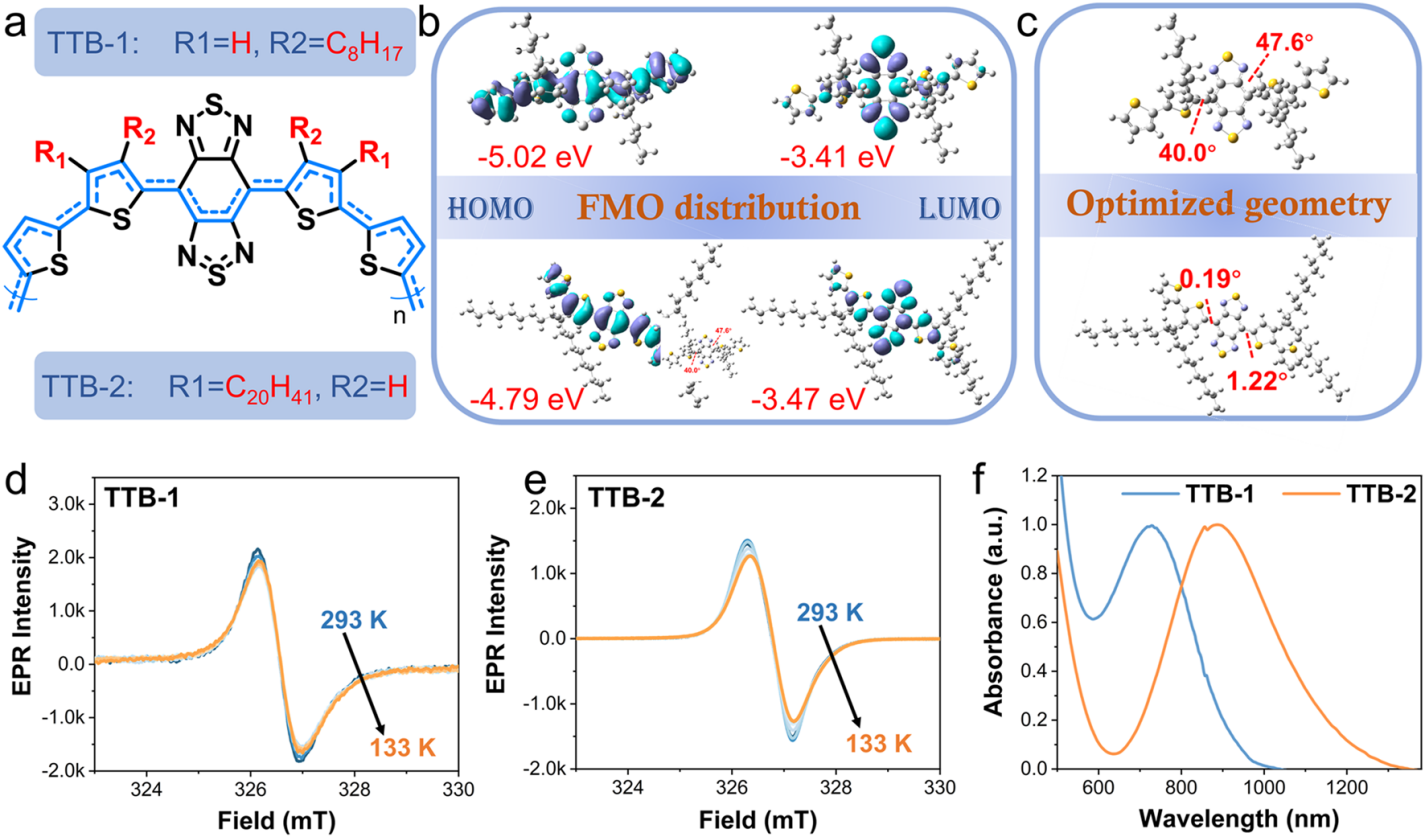
**a** Molecular structure of TTB. **b** FMO distributions of TTB-1 and TTB-2 calculated by DFT. **c** Orbital intramolecular torsional angles of TTB-1 and TTB-2. **d, e** ESR of **d** TTB-1 and **e** TTB-2 powder. **f** UV–Vis-NIR absorption spectra of TTB-1 and TTB-2 in THF

To achieve the above goal, two conjugated diradical polymer (CDPs) with a typical donor (D)-acceptor (A) configuration were synthesized by Stille polymerization of a strong electron-withdrawing benzo[1,2-c:4,5-c’]bis ([1,2,5]thiadiazole) (BBT) and electron-donating alkylthiophene (Fig. 1a). The detailed synthetic routes and the spectra of ^1^H NMR are added in Figs. S1–S4. The main difference between the two resulting conjugated diradical polymers is that 2-alkylthiophene and 3-alkylthiophene were separately arranged into the backbone of conjugated diradical polymers TTB-1 and TTB-2. For further physicochemical property demonstration, the density functional theory (DFT) was employed to calculate the electron distributions and optimized configurations of the two CDPs. As shown in Fig. 1b, there are pretty clear differences between the HOMO–LUMO (HOMO, highest occupied molecular orbital; LUMO, lowest unoccupied molecular orbital) energy gap of TTB-1 and TTB-2. The energy gap of TTB-2 is 1.32 eV, which is much lower than that of TTB-1 (1.62 eV). The main reason is that compared with TTB-1, TTB-2 has the distinct advantage of conjugated planar construction and intramolecular charge transfer, which possibly originate from the smaller steric hindrance caused by the position of the alkyl chain (Fig. 1c). As expected, the electron spinning resonance (ESR) spectroscopy demonstrated the good diradical feature of TTB-1 and TTB-2 with a g value equal to 2.0036 (Fig. 1d, e). ESR measurements of TTB at variable temperatures show a relatively invariant signal of spinning resonance, indicating good stabilities. Additionally, the absorption spectra of two TTB in THF solvent show that the TTB-1 has good absorption peaks at about 750 nm, while that of TTB-2 located at nearly 950 nm with almost 200 nm red-shift performance (Fig. 1f), which is consistent with the above DFT results.

### Photothermal Performance Characterization

**Fig. 2:**
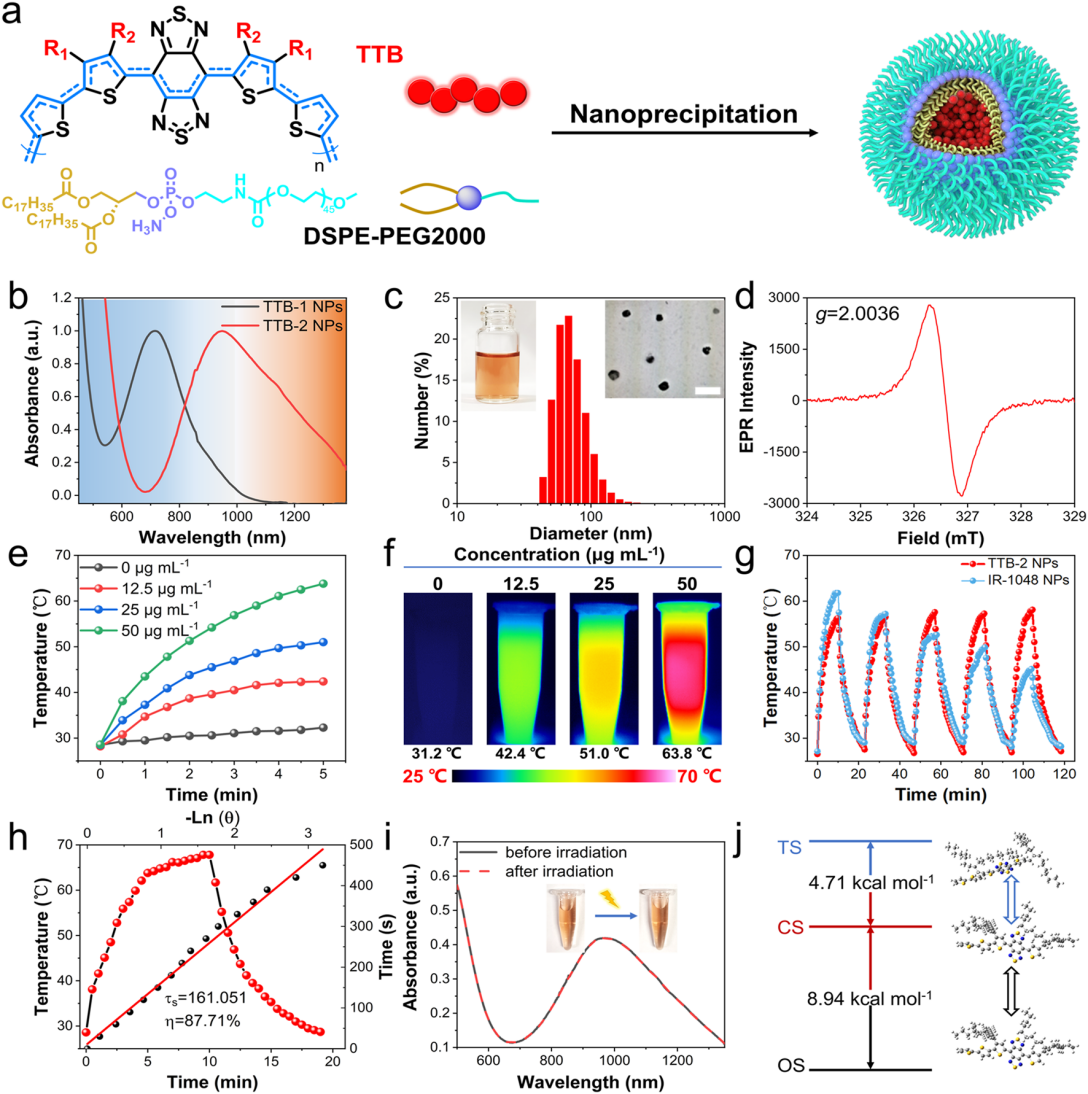
**a** Schematic illustration of the NPs preparation. **b** UV–Vis-NIR absorption spectra of TTB-1 NPs and TTB-2 NPs. **c** Representative size distribution of TTB-2 NPs and the insert is TEM image of TTB-2 NPs, scale bar: 200 nm. **d** ESR spectrum of TTB-2 NPs. **e** Heating curves and **f** infrared thermal imaging of TTB-2 NPs with different concentrations upon 1064 nm illumination. **g** Photothermal stability of 25 μg mL^−1^ TTB-2 NPs and 50 μg mL^−1^ IR-1048 NPs upon five on/off cycles of 1064 nm laser illumination (1.0 W cm^−2^). **h** Temperature change of TTB-2 NPs aqueous solution after 10 min illumination of 1064 nm laser (1.0 W cm^−2^) and then cooling, with a plot of time against the negative natural logarithm of temperature change during the cooling process. **i** UV–Vis-NIR absorption spectra and photographs of TTB-2 NPs before and after irradiation. **j** The energy levels of TTB-2 at the triplet state (TS), closed-shell singlet state (CS), and open-shell singlet state (OS)

To achieve good water-dispersive and long-circulation properties, the conjugated diradical polymers were prepared into nanoparticles by a self-assembly method using amphiphilic polymer DSPE-mPEG2000 as a coating agent (Fig. 2a). The UV–Vis-NIR absorption measurements show that TTB-2 NPs have the highest absorption peak at 950 nm with a tail beyond 1400 nm, while the maximum absorption of TTB-1 NPs locates at 715 nm (Fig. 2b). It is worth noting TTB-2 NPs clearly exhibit a typically strong absorption capability in the NIR- II window. Next, the countenance and size of the resulting NPs were characterized by transmission electron microscope (TEM) and dynamic light scattering (DLS) techniques. Both two NPs exhibit typical spherocytes with good uniformity in TEM imaging, and the hydrodynamic diameter of TTB-1 NPs are around 50 nm, while that of TTB-2 NPs are about 80 nm (Figs. 2c and S5). The ESR measurement further demonstrates that TTB-2 NPs aqueous solution remains an obvious diradical feature that is similar to the TTB-2 powder (Fig. 2d). These results indicate that the resulting TTB-2 NPs is a diradical NPs with NIR- II absorption properties. Next, the photothermal performances of TTB NPs were detailed characterized under the irradiation of a 1064-nm laser. As shown in Fig. 2e, the temperature of TTB-2 NPs aqueous solution at a concentration of 50 μg mL^−1^ rises rapidly upon 1064 nm laser irradiation, and finally arrives at 63.8 °C after 5 min irradiation. The temperature changes of TTB-2 NPs exhibit typical laser intensity-dependent capability (Fig. S6). The infrared thermal imaging further confirmed the good photothermal performances of TTB-2 NPs by Fig. 2f. Notably, TTB-2 NPs hold relatively high stability in the photothermal conversion after 5 on–off cycles of laser heating as shown in Fig. 2g compared with IR1048 NPs. There are negligible changes in the absorption spectrum, size and ESR spectroscopy of the TTB-2 NPs after laser irradiation, indicating their good photothermal stability (Figs. 2i and S7–S8). According to the calculation method in previous reports, the photothermal conversion efficiency (PCE) of the TTB-2 NPs are determined to be around 87.7%, which is far beyond the recently reported NIR-II PTT agents. (Fig. 2h and Table S1). As demonstrated in Fig. 2j, the excitation energy of the open-shell singlet state (OS) is 8.94 kcal mol^−1^ lower than the closed-shell singlet state (CS), implying that the OS state of TTB-2 typically tends to have good nonradiative decay property as noted by the energy gap law. Additionally, the TTB-2 NPs can sustain 28 days of stability when stored at 4 °C (Fig. S9). And, the ESR spectroscopy of TTB-2 NPs shows good stability after storing for 7 days (Fig. S10). As noted, the TTB-1 NPs show similar and stable photothermal properties with a PCE of 57. 6% upon 808 nm laser irradiation (Figs. S11-S16). TTB-2 NPs has almost no detectable fluorescence potential (Fig. S17). The NIR-II PA imaging properties of TTB-2 NPs were recorded at different concentrations (0 ~ 2.0 mg mL^−1^) (Fig. S18). Nanoparticles concentration-dependent PA intensity clarified the potential of TTB-2 NPs in photoacoustic imaging-guided photothermal therapy. As demonstrated, the TTB-2 NPs are unable to produce reactive oxygen species (ROS) under 1064 nm laser irradiation (Fig. S19). Furthermore, conjugated diradical polymer must be an important orientation in the library of NIR-II photothermal agents, and the next development of radical photothermal materials should focus on the combination of the other therapeutic method.

### In Vitro Cytotoxicity Assay

**Fig. 3:**
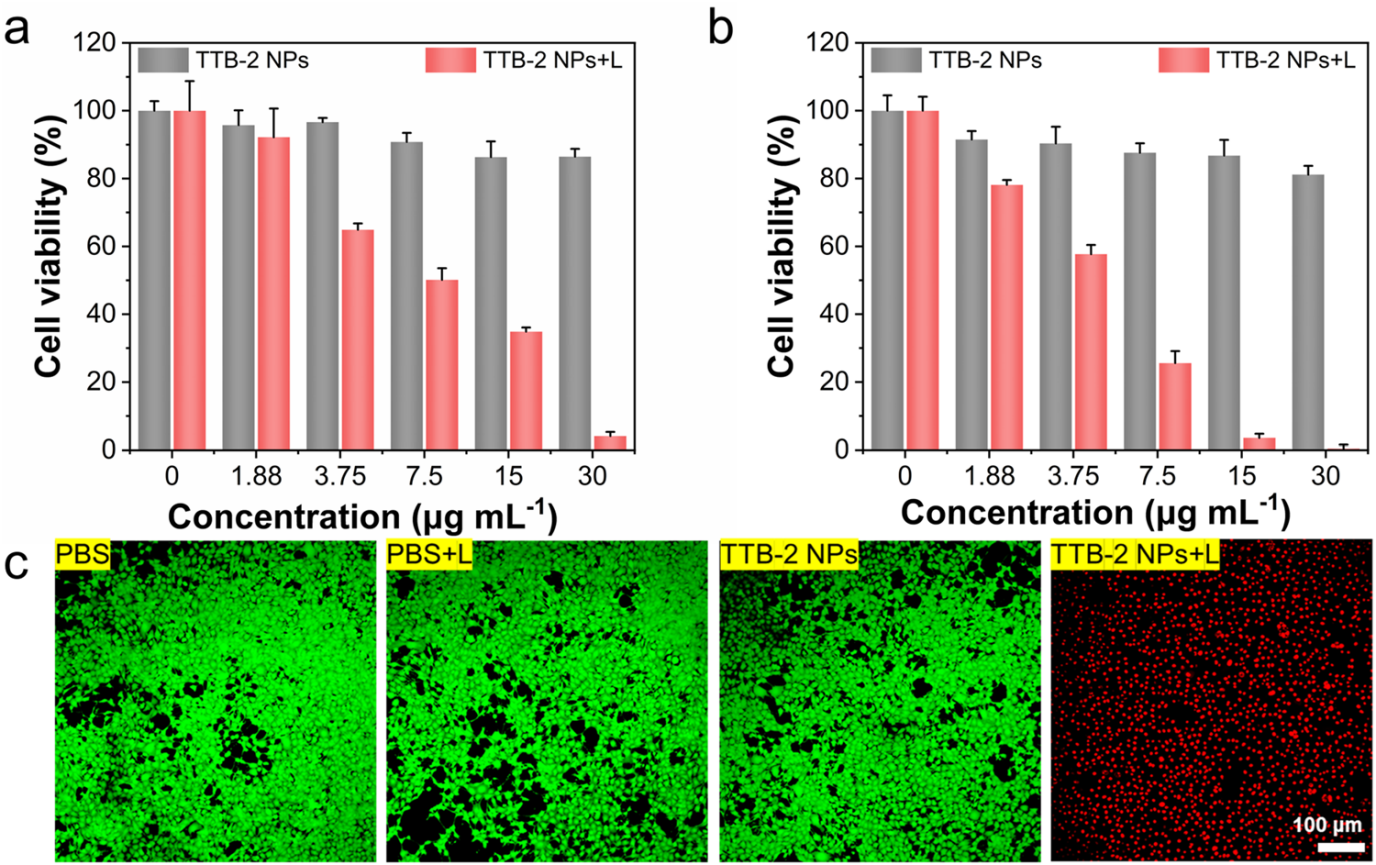
In vitro photothermic ablation of **a** HeLa and **b** 4T1 cells after being treated with TTB-2 NPs with or without 1064 nm irradiation (1.0 W cm^−2^) for 8 min. **c** Live/dead imaging of Calcein AM (live cells, green fluorescence) and PI (dead cells, red fluorescence) co-staining 4T1 cells with various treatment conditions

Motivated by the good photothermal performance and stability of TTB-2 NPs, we further evaluated in vitro anti-tumor photoablation performance. As shown in Figs. 3a and S20a, the TTB-2 NPs-induced significant photothermal ablation against the human cervical carcinoma (HeLa) cells upon 1064 nm laser irradiation, and nearly 95% of cells were completely killed at the concentration of TTB-2 NPs of 30 μg mL^−1^. In contrast, TTB-2 NPs only show slight damage toward cells without laser irradiation, suggesting good biocompatibility. Similar photoablation performances were also achieved in the murine breast cancer (4T1) cells (Figs. 3b and S20b). And also, TTB-2 NPs showed a slight cytotoxicity toward normal cells L929 at the concentration of 30 μg mL^−1^ (Fig. S21). Furthermore, the photoablation effect of TTB-2 NPs was further verified by the living/dead staining experiment (green fluorescent Calcein AM staining represents live cells, and red fluorescent PI staining indicates dead cells). As shown in Fig. 3c, all cells treated with TTB-2 NPs alone and laser only exhibited bright green fluorescence, proving that this has no effect on cell survival. However, after TTB-2 NPs treatment and laser irradiation, the cells appeared almost completely red fluorescence, which demonstrated that treated cells have been completely photoablation. These results demonstrate that TTB-2 NPs have good photoablation performance and thus hold potential for further in vivo applications.

### In Vivo PA Imaging and Photothermal Therapy

**Fig. 4:**
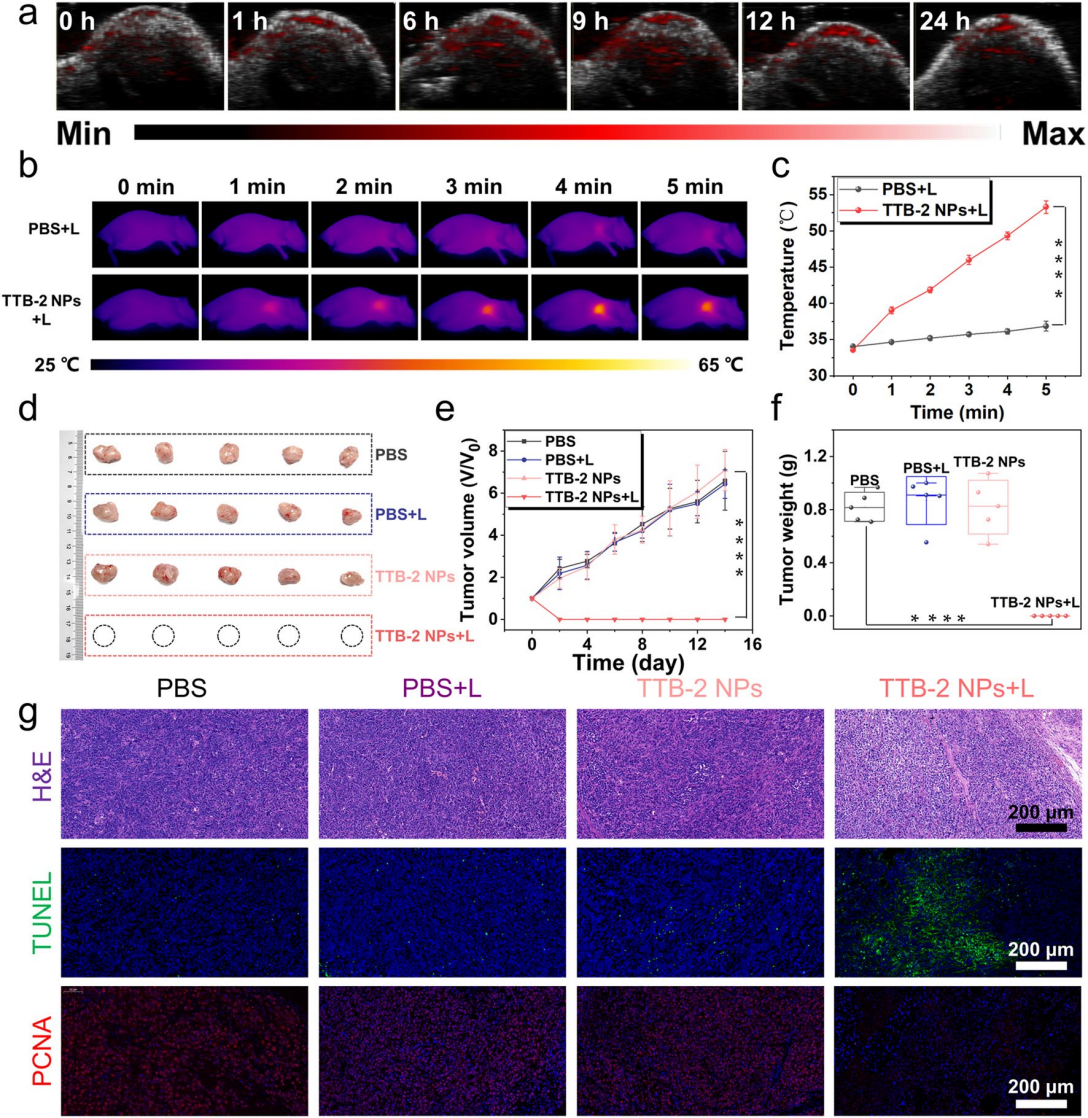
**a** In vivo PA imaging toward tumor at different times of TTB-2 NPs post-injection. **b** Representative thermal images and **c** corresponding temperature profile of tumor in various treated mice. **d** Tumor isolated from the mice after various treatments. **e** Relative tumor growth curves of different treatments as a function of treated time. **f** Tumor weight changes of different treatments at 14 days. **g** H&E, TUNEL, and PCNA staining of tumors separated from the treated mice. (**** *P* < 0.0001, *n* = 5)

Encouraged by the good in vitro photothermal properties of TTB-2 NPs, we further verified the in vivo anti-tumor performances. For photoacoustic (PA) imaging, the 4T1 tumor-xenografted mice were intravenously injected with TTB-2 NPs and then imaged by the PA imaging system. As shown in Figs. 4a and S22, the signals of TTB-2 NPs gradually appeared in the tumor location of 4T1 tumor-xenografted mice after intravenous injection, and then displayed an obvious peak at 9 h post-injection. After 24 h post-injection, TTB-2 NPs were almost entirely eliminated from the tumor site. According to the guidance of PA imaging, the optimal time for photothermal treatment was eventually identified at 9 h of post-injection. Next, the mice treated with various treatments were visualized the temperature change with an infrared thermal imager. As shown in Fig. 4b, c, the tumor temperature of the mice treated with TTB-2 NPs and 1064 nm irradiation is rapidly increased to 53 ℃ within 5 min laser irradiation, while that of the mice with laser irradiation only has a slight change. These results indicate that TTB-2 NPs can photothermally trigger efficient temperature changes in the tumor in vivo.

To further evaluate in vivo photoablation performance of TTB-2 NPs, the 4T1 tumor-xenografted mice with a tumor volume of around 80 mm^3^ were randomly divided into four groups. For the treatment group, the mice treated with TTB-2 NPs were irradiated with a 1064-nm laser for 5 min at the time of 9 post-injection, and then the tumor volume was monitored every other day. As shown in Fig. 4d-f, the tumor of the TTB-2 NPs-treated mice displayed nearly complete ablation on the second day after treatment, and there is no evidence of any recurrence in the 14 d periods of post-treatment. In contrast, there is no significant effect on tumor growth after TTB-2 NPs treatment alone and laser irradiation only. We also investigated the potential therapeutic mechanism of the photoablation of TTB-2 NPs by tumor biopsy. Hematoxylin and eosin (H&E) staining of tumors from the treated mice show that the photothermal activity of TTB-2 NPs triggered obvious mitosis and necrosis in the tumor site. Moreover, TUNEL and PCNA testing indicates that the photoablation effect of TTB-2 NPs indeed promoted significant tumor apoptosis and depressant proliferation in the tumor tissue. These results indicate that the TTB-2 NPs can efficiently locate in tumors and have a high therapeutic effect on in vivo tumors.

### Biosecurity of the TTB-2 NPs

**Fig. 5:**
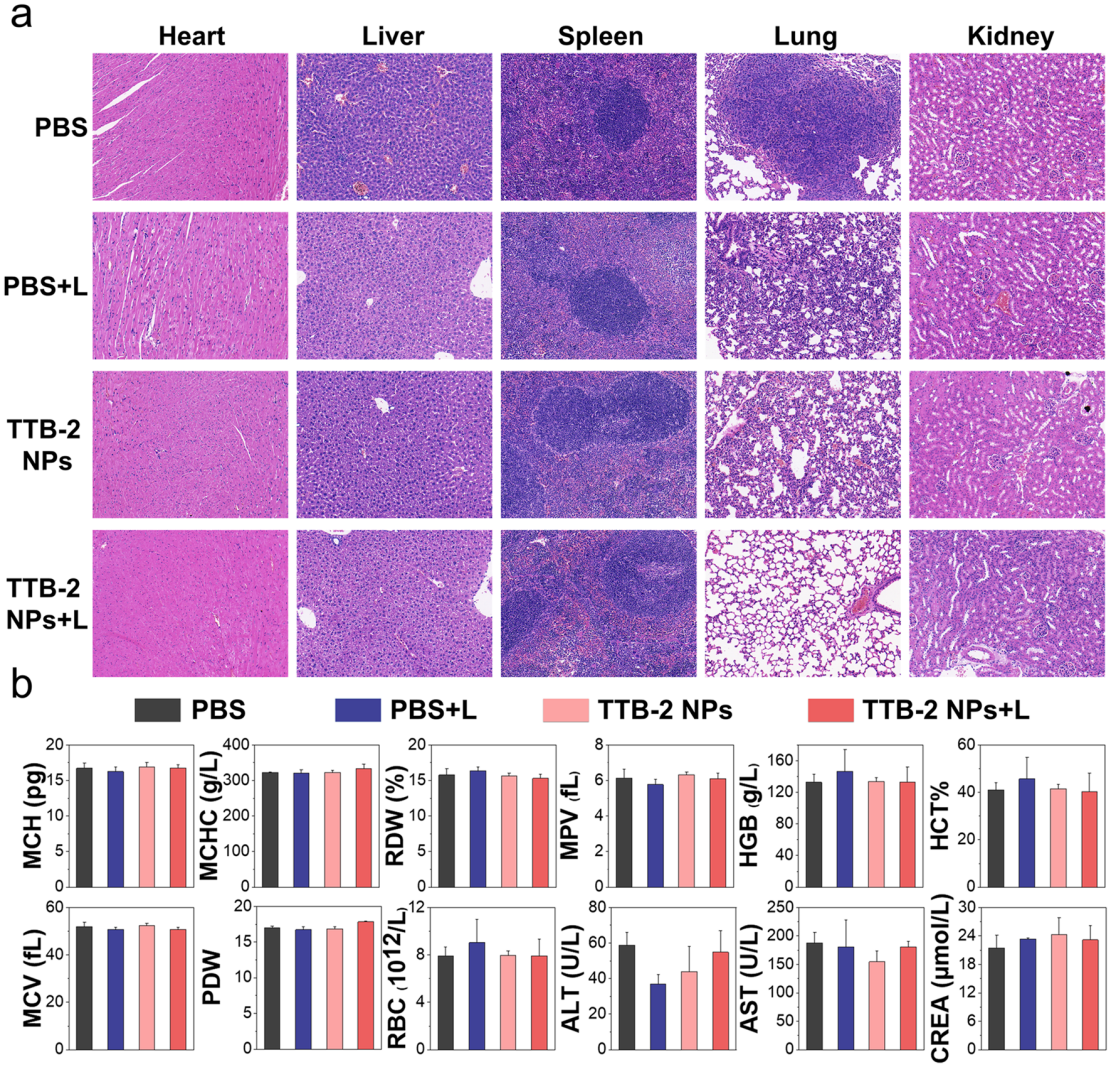
**a** H&E staining of main organs separated from the treated mice, scale bar: 50 μm. **b** Hematology markers and biochemical indexes analysis of mice in different groups

The preliminary biosecurity of the TTB-2 NPs was further studied after 14 days of treatments. No obvious change was clearly observed between the treated groups and the control group within 14 days of treatment (Fig. S24). The main organs mainly including the heart, lung, spleen, lung, and kidney collected from the mice with 14 days of treatments were analyzed by H&E staining and the results show that there is no obvious abnormality in the main organs (Fig. 5a). Next, the main hematology markers and blood biochemical parameters were examined and the results indicate that TTB-2 NPs treatment and laser irradiation have no apparent effect on these physiological parameters (Fig. 5b). The above results clearly proved the relatively high biosecurity of the TTB-2 NPs.

### In Vivo Photothermal Therapy Combined with Immunotherapy

**Fig. 6:**
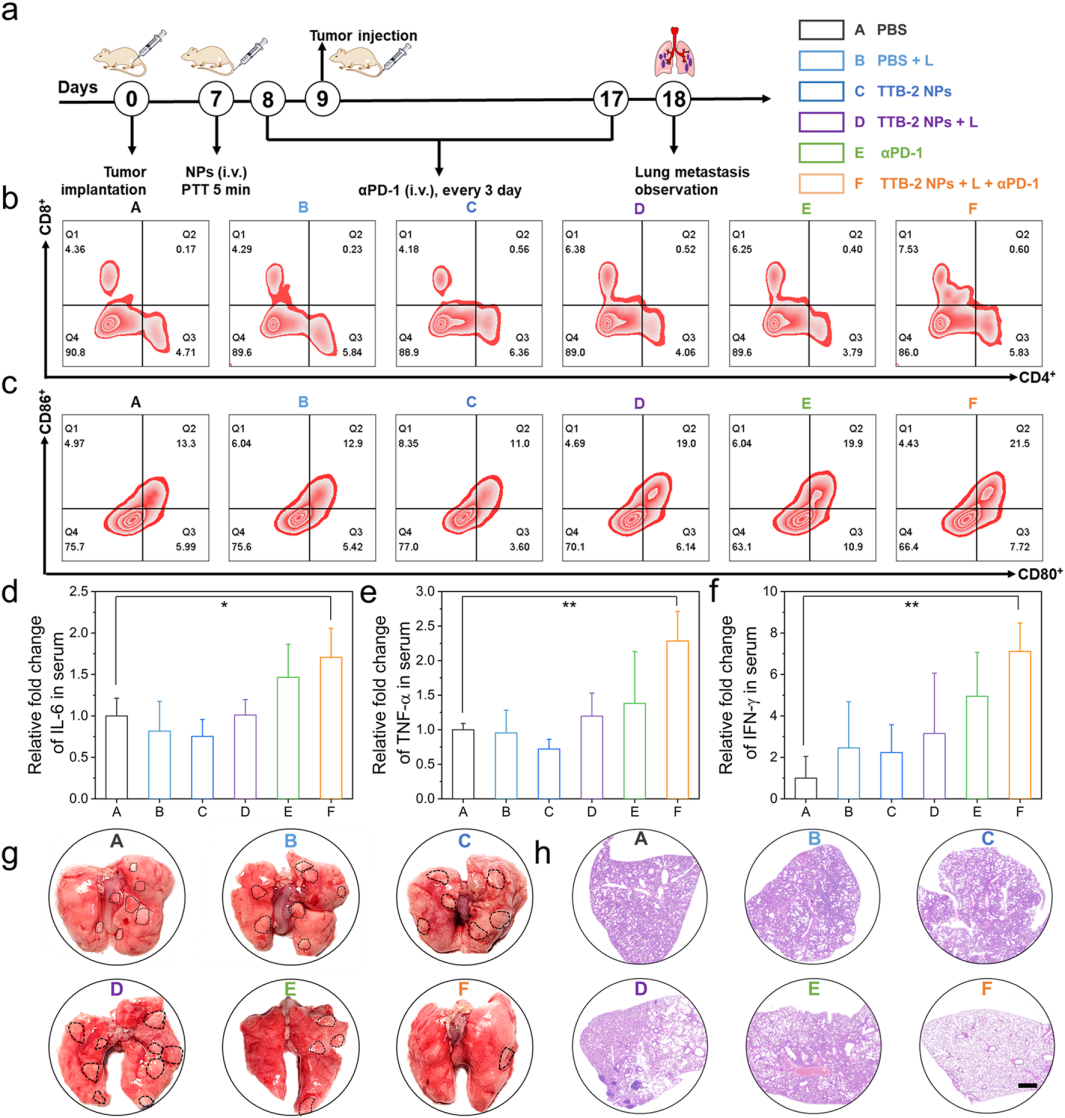
**a** Schematic diagram of the treatment schedule of anti-metastasis tumor treatment. **b** Flow cytometry analysis of CD8^+^ T cells (CD3^+^ CD8^+^) in tumor after different treatments. **c** CD80^+^ CD86^+^ T cells analysis in tumors from 4T1 tumor-bearing mice after different treatments by flow cytometry. ELISA assay of **d** IL-6, **e** TNF-α, and **f** IFN-γ levels in the tumors after different treatments. (*P < 0.05, ** P < 0.01, *n* = 3.) **g** Representative images of the whole lung. **h** H&E-staining of lung tissues. Scale bar: 500 μm

Furthermore, the anti-metastasis activity of TTB-2 NPs was evaluated by combined with αPD-1 (anti-PD1 antibody) in an aggressive tumor model with lung metastasis. The therapeutic procedure and parameters were clearly illustrated in Fig. 6a. Mice were treated with a PTT of TTB-2 NPs on the 7th day after subcutaneous tumor transplantation. After that, an anti-programmed death-1 antibody (αPD-1) (5 mg kg^−1^) was injected every 3 days. The 4T1 cells were intravenously injected into the above-treated mice on the 9th day, and mice of different groups were euthanized for metastasis analysis at 18 days of post-treatment. The TTB-2 NPs + L and TTB-2 NPs + L + αPD-1 can efficiently suppress and eliminate the subcutaneous tumors, while other groups including αPD-1 alone have almost no obvious therapeutic effect (Figs. S25 and S26). Furthermore, CD8^+^ cytotoxic T lymphocytes (CTLs) and mature dendritic (DC) cells (CD80^+^CD86^+^) of the tumor were examined by flow cytometry after the combination therapy of TTB-2 NPs PTT and αPD-1 immunotherapy and found that the combination therapy of TTB-2 NPs PTT and αPD-1 immunotherapy achieved much higher cytotoxic T cell (7.53%) and DC maturation levels (21.5%) than the PBS group with 4.36% of cytotoxic T cell and 13.3% of DC maturation (Figs. 6b, c and S28). And also, the mice treated with TTB-2 NPs PTT have obviously increased the level of cytotoxic T cell and DC maturation, suggesting TTB-2 NPs PTT and its combination therapy with αPD-1 immunotherapy can promote DCs maturation and boost systemic immune response. To further confirm the immune response, the enzyme-linked immunosorbent assays (ELISA) were further performed to test the level of cytokines and pro-inflammatory mediators in serum. As shown in Fig. 6d-f, the serum levels of IL-6, TNF-α, and IFN-γ in the mice after combination therapy of TTB-2 NPs PTT and αPD-1 immunotherapy were 1.7-fold, 2.0-fold, and 7.1-fold higher than those in PBS group, which is being significantly higher than that of αPD-1 treatment. These results clearly indicate that TTB-2 NPs PTT combined with αPD-1 immunotherapy can effectively promote DCs maturation to boost systemic immune response. In this evaluation, there still is a lack of investigation in the phenotypes (activation and exhaustion markers) of CD8^+^ T cells reflecting immunogenic or exhausted and the memory T cells of long-term antitumor immune memory, which is critical to know how the immune-environment was changed.

The pulmonary metastasis tumor was further analyzed after various treatments and illustrated in Fig. 6g. The lungs of the mice after PBS, PBS + L, TTB-2 NPs, TTB-2 NPs + L, and αPD-1 treatments have evident white nodules of metastatic tumor, while that the mice after the combination therapy of TTB-2 NPs PTT and αPD-1 immunotherapy clearly suppressed the metastatic tumor in the lung. H&E staining of the lung further confirms the above results (Fig. 6h). These results demonstrate that TTB-2 NPs PTT and its combination therapy with αPD-1 immunotherapy can efficiently activate the immune response for anti-metastasis tumor treatment.

## Conclusions

In conclusion, we demonstrated diradical-characterized NIR-II conjugated polymers as a superior PTT material for highly efficient photothermal immunotherapy of metastatic tumors. By engineering the side-chain position of conjugated diradical polymers, the absorption of biradical TTB-2 enables extending into the NIR-II region. Based on superhigh NIR-II photothermal conversion efficiency of 87.7% which is far beyond the reported PTT agents, we further demonstrated that TTB-2 NPs can achieve good tumor suppression and photoacoustic (PA) imaging performance in vitro and in vivo upon NIR-II light illustration. Moreover, combined with PD-1 treatment, TTB-2 NPs realize an efficient photo-immunity effect for suppressing pulmonary metastasis of breast cancer in vivo. Therefore, we ensure such conjugated diradical polymer must enrich the photothermal agents’ library in the NIR-II window and also provide helpful insight for the development of diradical materials.

## Supplementary Information

Below is the link to the electronic supplementary material.

Supplementary file1 (DOCX 9501 KB)

## References

[1] J. Li, L. Xie, B. Li, C. Yin, G. Wang et al., Engineering a hydrogen-sulfide-based nanomodulator to normalize hyperactive photothermal immunogenicity for combination cancer therapy. Adv. Mater. **33**(22), e2008481 (2021). 10.1002/adma.202008481 33899283 10.1002/adma.202008481

[2] Y. Jiang, J. Huang, C. Xu, K. Pu, Activatable polymer nanoagonist for second near-infrared photothermal immunotherapy of cancer. Nat. Commun. **12**(1), 742 (2021). 10.1038/s41467-021-21047-0 33531498 10.1038/s41467-021-21047-0 PMC7854754

[3] Y. Li, Y. Tang, W. Hu, Z. Wang, X. Li et al., Incorporation of robust NIR-II fluorescence brightness and photothermal performance in a single large π-conjugated molecule for phototheranostics. Adv. Sci. **10**(3), 2204695 (2022). 10.1002/advs.202204695 10.1002/advs.202204695 PMC9875648 36453572

[4] K. Wang, Y. Li, X. Wang, Z. Zhang, L. Cao et al., Gas therapy potentiates aggregation-induced emission luminogen-based photoimmunotherapy of poorly immunogenic tumors through cgas-sting pathway activation. Nat. Commun. **14**(1), 2950 (2023). 10.1038/s41467-023-38601-7 37221157 10.1038/s41467-023-38601-7 PMC10205712

[5] J. Li, Y. Luo, K. Pu, Electromagnetic nanomedicines for combinational cancer immunotherapy. Angew. Chem. Int. Ed. **60**(23), 12682–12705 (2021). 10.1002/anie.202008386 10.1002/anie.202008386 32671893

[6] X. Wei, C. Zhang, S. He, J. Huang, J. Huang et al., A dual-locked activatable phototheranostic probe for biomarker-regulated photodynamic and photothermal cancer therapy. Angew. Chem. Int. Ed. **61**(26), e202202966 (2022). 10.1002/anie.202202966 10.1002/anie.202202966 35396786

[7] X. Hu, N. Wang, X. Guo, Z. Liang, H. Sun et al., A sub-nanostructural transformable nanozyme for tumor photocatalytic therapy. Nano-Micro Lett. **14**, 101 (2022). 10.1007/s40820-022-00848-y 10.1007/s40820-022-00848-y PMC9005554 35412159

[8] C. Xu, Y. Jiang, Y. Han, K. Pu, R. Zhang, A polymer multicellular nanoengager for synergistic NIR-II photothermal immunotherapy. Adv. Mater. **33**(14), e2008061 (2021). 10.1002/adma.202008061 33634897 10.1002/adma.202008061

[9] Y. Xia, S. Fu, Q. Ma, Y. Liu, N. Zhang, Application of nano-delivery systems in lymph nodes for tumor immunotherapy. Nano-Micro Lett. **15**, 145 (2023). 10.1007/s40820-023-01125-2 10.1007/s40820-023-01125-2 PMC10239433 37269391

[10] H. Feng, Y. Yuan, Y. Zhang, H.-J. Liu, X. Dong et al., Targeted micellar phthalocyanine for lymph node metastasis homing and photothermal therapy in an orthotopic colorectal tumor model. Nano-Micro Lett. **13**, 145 (2021). 10.1007/s40820-021-00666-8 10.1007/s40820-021-00666-8 PMC8214644 34146159

[11] Z. Huang, Y. Liu, S. Li, C.-S. Lee, X.H. Zhang, From materials to devices: rationally designing solar steam system for advanced applications. Small Methods **6**(10), e2200835 (2022). 10.1002/smtd.202200835 36100465 10.1002/smtd.202200835

[12] J. Zhang, Y. Li, M. Jiang, H. Qiu, Y. Li et al., Self-assembled aza-bodipy and iron(iii) nanoparticles for photothermal-enhanced chemodynamic therapy in the NIR-II window. ACS Biomater. Sci. Eng. **9**(2), 821–830 (2023). 10.1021/acsbiomaterials.2c01539 36725684 10.1021/acsbiomaterials.2c01539

[13] N. Song, Z. Zhang, P. Liu, D. Dai, C. Chen et al., Pillar[5]arene-modified gold nanorods as nanocarriers for multi-modal imaging-guided synergistic photodynamic-photothermal therapy. Adv. Funct. Mater. **31**(21), 2009924 (2021). 10.1002/adfm.202009924

[14] M. Fu, Y. Shen, H. Zhou, X. Liu, W. Chen et al., Gallium-based liquid metal micro/nanoparticles for photothermal cancer therapy. J. Mater. Sci. Technol. **142**, 22–33 (2023). 10.1016/j.jmst.2022.08.049

[15] B. Chen, C. Zhang, W. Wang, Z. Chu, Z. Zha et al., Ultrastable AgBis(2) hollow nanospheres with cancer cell-specific cytotoxicity for multimodal tumor therapy. ACS Nano **14**(11), 14919–14928 (2020). 10.1021/acsnano.0c04370 33137257 10.1021/acsnano.0c04370

[16] S. Xiao, Y. Lu, M. Feng, M. Dong, Z. Cao et al., Multifunctional FeS_2_ theranostic nanoparticles for photothermal-enhanced chemodynamic/photodynamic cancer therapy and photoacoustic imaging. Chem. Eng. J. **396**, 125294 (2020). 10.1016/j.cej.2020.125294

[17] M. Liu, H. Zhu, Y. Wang, C. Sevencan, B.L. Li, Functionalized MoS_2_-based nanomaterials for cancer phototherapy and other biomedical applications. ACS Mater. Lett. **3**(5), 462–496 (2021). 10.1021/acsmaterialslett.1c00073

[18] Y. Zhao, M. Song, X. Yang, J. Yang, C. Du et al., Amorphous Ag_2_-xCuxS quantum dots: “All-in-one” theranostic nanomedicines for near-infrared fluorescence/photoacoustics dual-modal-imaging-guided photothermal therapy. Chem. Eng. J. **399**, 125777 (2020). 10.1016/j.cej.2020.125777

[19] G. Xu, X. Bao, J. Chen, B. Zhang, D. Li et al., In vivo tumor photoacoustic imaging and photothermal therapy based on supra-(carbon nanodots). Adv. Healthc. Mater. **8**(2), e1800995 (2019). 10.1002/adhm.201800995 30474227 10.1002/adhm.201800995

[20] C. Yang, M.R. Younis, J. Zhang, J. Qu, J. Lin et al., Programmable NIR-II photothermal-enhanced starvation-primed chemodynamic therapy using glucose oxidase-functionalized ancient pigment nanosheets. Small **16**(25), e2001518 (2020). 10.1002/smll.202001518 32468633 10.1002/smll.202001518

[21] S.W. Jun, P. Manivasagan, J. Kwon, V.T. Nguyen, S. Mondal et al., Folic acid-conjugated chitosan-functionalized graphene oxide for highly efficient photoacoustic imaging-guided tumor-targeted photothermal therapy. Int. J. Biol. Macromol. **155**, 961–971 (2020). 10.1016/j.ijbiomac.2019.11.055 31712157 10.1016/j.ijbiomac.2019.11.055

[22] P. Sun, X. Jiang, B. Sun, H. Wang, J. Li et al., Electron-acceptor density adjustments for preparation conjugated polymers with NIR-II absorption and brighter NIR-II fluorescence and 1064 nm active photothermal/gas therapy. Biomaterials **280**, 121319 (2022). 10.1016/j.biomaterials.2021.121319 34923313 10.1016/j.biomaterials.2021.121319

[23] X. Wang, X. Wang, Q. Yue, H. Xu, X. Zhong et al., Liquid exfoliation of tin nanodots as novel sonosensitizers for photothermal-enhanced sonodynamic therapy against cancer. Nano Today **39**, 101170 (2021). 10.1016/j.nantod.2021.101170

[24] S. Hao, H. Han, Z. Yang, M. Chen, Y. Jiang et al., Recent advancements on photothermal conversion and antibacterial applications over mxenes-based materials. Nano-Micro Lett. **14**, 178 (2022). 10.1007/s40820-022-00901-w 10.1007/s40820-022-00901-w PMC9402885 36001173

[25] B. Guo, Z. Sheng, D. Hu, C. Liu, H. Zheng et al., Through scalp and skull NIR-II photothermal therapy of deep orthotopic brain tumors with precise photoacoustic imaging guidance. Adv. Mater. **30**(35), e1802591 (2018). 10.1002/adma.201802591 30129690 10.1002/adma.201802591

[26] Y. Wan, G. Lu, W.C. Wei, Y.H. Huang, S.L. Li et al., Stable organic photosensitizer nanoparticles with absorption peak beyond 800 nanometers and high reactive oxygen species yield for multimodality phototheranostics. ACS Nano **14**(8), 9917–9928 (2020). 10.1021/acsnano.0c02767 32706236 10.1021/acsnano.0c02767

[27] M. Li, Z. Li, D. Yu, M. Wang, D. Wang et al., Quinoid conjugated polymer nanoparticles with NIR-II absorption peak toward efficient photothermal therapy. Chem. (2022). 10.1002/chem.202202930 10.1002/chem.202202930 36484147

[28] Y. Zou, W. Liu, W. Sun, J. Du, J. Fan et al., Highly inoxidizable heptamethine cyanine–glucose oxidase conjugate nanoagent for combination of enhanced photothermal therapy and tumor starvation. Adv. Funct. Mater. **32**(17), 2111853 (2022). 10.1002/adfm.202111853

[29] J. Wu, Y. Zhang, K. Jiang, X. Wang, N.T. Blum et al., Enzyme-engineered conjugated polymer nanoplatform for activatable companion diagnostics and multistage augmented synergistic therapy. Adv. Mater. **34**(18), e2200062 (2022). 10.1002/adma.202200062 35243699 10.1002/adma.202200062

[30] D. Zheng, P. Yu, Z. Wei, C. Zhong, M. Wu et al., RBC membrane camouflaged semiconducting polymer nanoparticles for near-infrared photoacoustic imaging and photothermal therapy. Nano-Micro Lett. **12**, 94 (2020). 10.1007/s40820-020-00429-x 10.1007/s40820-020-00429-x PMC7770914 34138120

[31] H. Zhou, D. Tang, X. Kang, H. Yuan, Y. Yu et al., Degradable pseudo conjugated polymer nanoparticles with NIR-II photothermal effect and cationic quaternary phosphonium structural bacteriostasis for anti-infection therapy. Adv. Sci. **9**(16), e2200732 (2022). 10.1002/advs.202200732 10.1002/advs.202200732 PMC9165483 35343113

[32] S. Li, Q. Deng, Y. Zhang, X. Li, G. Wen et al., Rational design of conjugated small molecules for superior photothermal theranostics in the NIR-II biowindow. Adv. Mater. **32**(33), e2001146 (2020). 10.1002/adma.202001146 32627868 10.1002/adma.202001146

[33] P. Chen, F. Qu, S. Chen, J. Li, Q. Shen et al., Bandgap modulation and lipid intercalation generates ultrabright D-A-D-based zwitterionic small-molecule nanoagent for precise NIR-II excitation phototheranostic applications. Adv. Funct. Mater. **32**(52), 202208463 (2022). 10.1002/adfm.202208463

[34] C. Zhang, M. Xu, Z. Zeng, X. Wei, S. He et al., A polymeric extracellular matrix nanoremodeler for activatable cancer photo-immunotherapy. Angew. Chem. Int. Ed. **62**(12), e202217339 (2023). 10.1002/anie.202217339 10.1002/anie.202217339 36694443

[35] D. Wang, J. Liu, C. Wang, W. Zhang, G. Yang et al., Microbial synthesis of prussian blue for potentiating checkpoint blockade immunotherapy. Nat. Commun. **14**(1), 2943 (2023). 10.1038/s41467-023-38796-9 37221237 10.1038/s41467-023-38796-9 PMC10205718

[36] Y. Qin, X. Chen, Y. Gui, H. Wang, B.Z. Tang et al., Self-assembled metallacage with second near-infrared aggregation-induced emission for enhanced multimodal theranostics. J. Am. Chem. Soc. **144**(28), 12825–12833 (2022). 10.1021/jacs.2c03895 35786928 10.1021/jacs.2c03895

[37] P. Xiao, W. Xie, J. Zhang, Q. Wu, Z. Shen et al., De novo design of reversibly PH-switchable NIR-II aggregation-induced emission luminogens for efficient phototheranostics of patient-derived tumor xenografts. J. Am. Chem. Soc. **145**(1), 334–344 (2023). 10.1021/jacs.2c10076 36575385 10.1021/jacs.2c10076

[38] D. Yan, M. Wang, Q. Wu, N. Niu, M. Li et al., Multimodal imaging-guided photothermal immunotherapy based on a versatile NIR-II aggregation-induced emission luminogen. Angew. Chem. Int. Ed. **61**(27), e202202614 (2022). 10.1002/anie.202202614 10.1002/anie.202202614 35344252

[39] J. Li, J. Wang, J. Zhang, T. Han, X. Hu et al., A facile strategy of boosting photothermal conversion efficiency through state transformation for cancer therapy. Adv. Mater. **33**(51), e2105999 (2021). 10.1002/adma.202105999 34651361 10.1002/adma.202105999

[40] R. Zheng, Q. Zhao, W. Qing, S. Li, Z. Liu et al., Carrier-free delivery of ultrasmall π-conjugated oligomer nanoparticles with photothermal conversion over 80% for cancer theranostics. Small **18**(4), e2104521 (2022). 10.1002/smll.202104521 34821029 10.1002/smll.202104521

[41] S. Chen, Y. Pan, K. Chen, P. Chen, Q. Shen et al., Increasing molecular planarity through donor/side-chain engineering for improved NIR-IIa fluorescence imaging and NIR-II photothermal therapy under 1064 nm. Angew. Chem. Int. Ed. **62**(6), e202215372 (2023). 10.1002/anie.202215372 10.1002/anie.202215372 36480198

[42] X. Wu, Y. Jiang, N.J. Rommelfanger, F. Yang, Q. Zhou et al., Tether-free photothermal deep-brain stimulation in freely behaving mice via wide-field illumination in the Near-Infrared-II window. Nat. Biomed. Eng. **6**(6), 754–770 (2022). 10.1038/s41551-022-00862-w 35314800 10.1038/s41551-022-00862-w PMC9232843

[43] C. Zhou, L. Zhang, T. Sun, Y. Zhang, Y. Liu et al., Activatable NIR-II plasmonic nanotheranostics for efficient photoacoustic imaging and photothermal cancer therapy. Adv. Mater. **33**(3), 2006532 (2020). 10.1002/adma.202006532 10.1002/adma.202006532 33283355

[44] D. Tang, H. Zhou, M. Cui, G. Liang, H. Zhang et al., NIR-II light accelerated prodrug reduction of Pt(iv)-incorporating pseudo semiconducting polymers for robust degradation and maximized photothermal/chemo-immunotherapy. Adv. Mater. **35**(28), 2300048 (2023). 10.1002/adma.202300048 10.1002/adma.202300048 37016274

[45] A. Abdurahman, T.J.H. Hele, Q. Gu, J. Zhang, Q. Peng et al., Understanding the luminescent nature of organic radicals for efficient doublet emitters and pure-red light-emitting diodes. Nat. Mater. **19**(11), 1224–1229 (2020). 10.1038/s41563-020-0705-9 32541936 10.1038/s41563-020-0705-9

[46] H. Guo, Q. Peng, X.K. Chen, Q. Gu, S. Dong et al., High stability and luminescence efficiency in donor-acceptor neutral radicals not following the aufbau principle. Nat. Mater. **18**(9), 977–984 (2019). 10.1038/s41563-019-0433-1 31332338 10.1038/s41563-019-0433-1

[47] X. Ai, E.W. Evans, S. Dong, A.J. Gillett, H. Guo et al., Efficient radical-based light-emitting diodes with doublet emission. Nature **563**(7732), 536–540 (2018). 10.1038/s41586-018-0695-9 30464267 10.1038/s41586-018-0695-9

[48] K. Oyaizu, H. Nishide, Radical polymers for organic electronic devices: A radical departure from conjugated polymers? Adv. Mater. **21**(22), 2339–2344 (2009). 10.1002/adma.200803554

[49] J. Kida, D. Aoki, H. Otsuka, Mechanophore activation enhanced by hydrogen bonding of diarylurea motifs: an efficient supramolecular force-transducing system. Aggregate. **2**(3), e50 (2021). 10.1002/agt2.50

[50] T. Janoschka, M.D. Hager, U.S. Schubert, Powering up the future: Radical polymers for battery applications. Adv. Mater. **24**(48), 6397–6409 (2012). 10.1002/adma.201203119 23238940 10.1002/adma.201203119

[51] W. Zeng, J. Wu, Open-shell graphene fragments. Chem **7**(2), 358–386 (2021). 10.1016/j.chempr.2020.10.009

[52] M. Slota, A. Keerthi, W.K. Myers, E. Tretyakov, M. Baumgarten et al., Magnetic edge states and coherent manipulation of graphene nanoribbons. Nature **557**(7707), 691–695 (2018). 10.1038/s41586-018-0154-7 29849157 10.1038/s41586-018-0154-7

[53] X. Hu, W. Wang, D. Wang, Y. Zheng, The electronic applications of stable diradicaloids: present and future. J. Mater. Chem. C **6**(42), 11232–11242 (2018). 10.1039/c8tc04484h

[54] L. Li, Y. Li, A study on the origin of the radical in fullerene and graphene. The J. Phys. Chem. C **122**(16), 8780–8787 (2018). 10.1021/acs.jpcc.8b00515

[55] Y. Li, L. Li, Y. Wu, Y. Li, A review on the origin of synthetic metal radical: Singlet open-shell radical ground state? J. Phys. Chem. C **121**(15), 8579–8588 (2017). 10.1021/acs.jpcc.6b12936

[56] J. Guo, C. Zhou, S. Xie, S. Luo, T.Y. Gopalakrishna et al., Large aromatic hydrocarbon radical cation with global aromaticity and state-associated magnetic activity. Chem. Mater. **32**(14), 5927–5936 (2020). 10.1021/acs.chemmater.9b05099

[57] Z. Chen, W. Li, M.A. Sabuj, Y. Li, W. Zhu et al., Evolution of the electronic structure in open-shell donor-acceptor organic semiconductors. Nat. Commun. **12**(1), 5889 (2021). 10.1038/s41467-021-26173-3 34620849 10.1038/s41467-021-26173-3 PMC8497548

[58] T. Luo, Y. Wang, J. Hao, P.A. Chen, Y. Hu et al., Furan-extended helical rylenes with fjord edge topology and tunable optoelectronic properties. Angew. Chem. Int. Ed. **62**(5), e202214653 (2023). 10.1002/anie.202214653 10.1002/anie.202214653 36470852

[59] Z. Chen, W. Li, Y. Zhang, Z. Wang, W. Zhu et al., Aggregation-induced radical of donor-acceptor organic semiconductors. J. Phys. Chem. Lett. **12**(40), 9783–9790 (2021). 10.1021/acs.jpclett.1c02463 34596405 10.1021/acs.jpclett.1c02463

[60] B. Tang, W.L. Li, Y. Chang, B. Yuan, Y. Wu et al., A supramolecular radical dimer: High-efficiency NIR-II photothermal conversion and therapy. Angew. Chem. Int. Ed. **58**(43), 15526–15531 (2019). 10.1002/anie.201910257 10.1002/anie.201910257 31478324

[61] B. Lu, Y. Chen, P. Li, B. Wang, K. Mullen et al., Stable radical anions generated from a porous perylenediimide metal-organic framework for boosting near-infrared photothermal conversion. Nat. Commun. **10**(1), 767 (2019). 10.1038/s41467-019-08434-4 30770818 10.1038/s41467-019-08434-4 PMC6377642

[62] Z. Mi, P. Yang, R. Wang, J. Unruangsri, W. Yang et al., Stable radical cation-containing covalent organic frameworks exhibiting remarkable structure-enhanced photothermal conversion. J. Am. Chem. Soc. **141**(36), 14433–14442 (2019). 10.1021/jacs.9b07695 31426635 10.1021/jacs.9b07695

[63] G. Chen, J. Sun, Q. Peng, Q. Sun, G. Wang et al., Biradical-featured stable organic-small-molecule photothermal materials for highly efficient solar-driven water evaporation. Adv. Mater. **32**(29), e1908537 (2020). 10.1002/adma.201908537 32519356 10.1002/adma.201908537

[64] X. Cui, G. Lu, S. Dong, S. Li, Y. Xiao et al., Stable π-radical nanoparticles as versatile photosensitizers for effective hypoxia-overcoming photodynamic therapy. Mater. Horiz. **8**(2), 571–576 (2021). 10.1039/d0mh01312a 34821273 10.1039/d0mh01312a

[65] X. Cui, Z. Zhang, Y. Yang, S. Li, C.-S. Lee, Organic radical materials in biomedical applications: state of the art and perspectives. Exploration **2**(2), 20210264 (2022). 10.1002/exp.20210264 37323877 10.1002/EXP.20210264 PMC10190988

