# Supplementary material for: A Stable Open-Shell Conjugated Diradical Polymer with Ultra-High Photothermal Conversion Efficiency for NIR-II Photo-Immunotherapy of Metastatic Tumor
Source: Nanomicro Lett. 2023 Nov 20;16:21. doi: 10.1007/s40820-023-01219-x (PMC10660627; doi:10.1007/s40820-023-01219-x)
Supplement: Supplementary file 1 — Supplementary file1 (DOCX 9501 KB) [file 40820_2023_1219_MOESM1_ESM.docx]

**Supplementary Information**

A Stable Open-Shell Conjugated Diradical Polymer with Ultra-High Photothermal Conversion Efficiency for NIR-II Photo-Immunotherapy of Metastatic Tumor

Yijian Gao^1^, Ying Liu^1^, Xiliang Li^1^, Hui Wang^2^, Yingpeng Wan^3^, Yuliang Yang^1^, Yu Luo^1^, Chun-sing Lee^3,^*, Shengliang Li, ^1,^* and Xiao-Hong Zhang^2,^*

^1^ College of Pharmaceutical Sciences, Soochow University, Suzhou 215123, P.R. China

^2^ Institute of Functional Nano & Soft Materials (FUNSOM), Soochow University, Suzhou 215123, P. R. China

^3^ Center of Super-Diamond and Advanced Films (COSDAF) & Department of Chemistry, City University of Hong Kong, 83 Tat Chee Avenue, Kowloon, Hong Kong SAR, P. R. China.

*Corresponding author. E-mail: [lishengliang@suda.edu.cn](mailto:lishengliang@suda.edu.cn); [xiaohong_zhang@suda.edu.cn](mailto:xiaohong_zhang@suda.edu.cn); [apcslee@cityu.edu.hk](mailto:apcslee@cityu.edu.hk).

S1 Experimental

**S1.1 Materials**

RPMI 1640 culturing media and fetal bovine serum were purchased from Thermo Fisher Scientific (China) Co., Ltd. LIVE/DEAD^TM^ Fixable Dead Cell Stain Kit (catalog: L34966) was purchased from Thermo Fisher Scientific (China) Co., Ltd. Anti-mouse-PD-1 antibodies (αPD-1, catalog: BE0146, clone: RMP1-14) were purchased from BioXcell Co., Ltd. ELISA kits for TNF-α, IFN-γ and IL-6 assay were all purchased from Aimeng youning (Shanghai, China). FITC anti-mouse CD3 (catalog: 100204), PE/Cyanine7 anti-mouse CD4 (catalog: 116016), APC/Cyanine7 anti-mouse CD8a (catalog: 100714), PE anti-mouse CD80 (catalog: 104708) and APC anti-mouse CD86 (catalog: 105012) antibodies were purchased from BioLegend, Inc. (San Diego, USA). Collagenase II (catalog: C8150) were purchased from Solarbio life sciences Co., Ltd. (Beijing, China).

**S1.2 Theoretical simulation of TTB**

 Density functional theory (DFT) calculations were performed by using Gaussian 09 with the B3LYP/6-31 g (d) basis set. The electrostatic potential is then calculated based on the optimized S_0_ molecular geometries at the same level and analyzed with Multiwfn 3.8 code. The excitation energies and oscillator strengths were calculated by timedependent DFT. The electronic structures and properties of the closed-shell state is evaluated by using restricted DFT and those of the open-shell state is computed by using unrestricted DFT.

**S1.3 Photothermal performance characterization**

An 808 nm NIR-I light was used to irradiate TTB-1 NPs and 1064 nm NIR-II light for TTB-2 NPs (0.5 mL). A Ti480 pro thermal imaging camera was used to examine the temperature change of TTB NPs with different concentrations (1.0 W cm^-2^, 0, 12.5, 25 and 50 μg mL^-1^) and different laser power intensities (50 μg mL^-1^,0.5, 0.75 and 1.0 W cm^-2^,). Observe 5 cycles of laser irradiation (1.0 W cm^-2^,) for 10 minutes and natural cooling to room temperature to verify the stability of TTB NPs (50 μg mL^-1^ for TTB-1 NPs and 25 μg mL^-1^ for TTB-2 NPs).

The calculation of photothermal conversion efficiency (η) is referred to the previous literature as following equation ^[1]^. The temperature profile of TTB-2 NPs (0.5 mL, 50 μg mL^-1^) is obtained under 1064 nm laser irradiation (1 W cm^-2^). When there is a plateau of temperature increase, the laser was turned off and the corresponding temperature was recorded every 30 s until the temperature returns to room temperature.

$\eta=\frac{hS\left( T_{Max}-T_{Surr} \right)-Q_{Dis}}{I\left( 1-{10}^{-A1064} \right)}$ ……(1)

where *h* is the heat transfer coefficient, *S* is container area, *T_max_* is the temperature difference between peak flat-state temperature and room temperature, *Q_Dis_* is the heat relevant to the light absorbance of DI water, *I* is the laser power density, and *A_1064_* is the absorption intensity of TTB-2 NPs at 1064 nm. *hS* can be obtained by equation (2).

$\tau_{s}=\frac{m_{D}C_{D}}{hS}$ …… (2)

where *m_D_* is the mass of pure water (0.5 g), and *C_D_* is the heat capacity of pure water (4.2 J/g).

τ_s_ can be obtained by equation (3).

$t=-\tau_{s}ln( )$ …… (3);

where *θ* is the ratio of is the ratio of *ΔT* to *ΔT_max_*. *ΔT* is the temperature difference between the cooling temperature every 30 s intervals and room temperature.

The same calculation method was used for PCE of TTB-1 NPs.

**S1.4 *In Vitro* Cytotoxicity Assay**

Mice breast cancer cells (4T1) and human cervical cancer cell (Hela) were provided by the Institute of Basic Medical Sciences, Chinese Academy of Medical Sciences (Beijing, China). 4T1 or Hela cells were inoculated into a 96-well cell culture plate (4 × 10^3^/ well), and various concentrations of TTB NPs (0, 1.88, 3.75, 7.50, 15.0, 30.0 μg mL^-1^) were added to incubate after the cell adherence. Change the culture medium after 4 hours, the NPs + Laser group was irradiated by NIR laser (1.0 W cm^-2^, 8 min). After incubation for 24 hours, 100 μL of MTT (0.5 mg mL^-1^) solution was added to each well in the dark. 100 μL of DMSO was added to each well to dissolve the purple crystal. The absorbance at 492 nm was obtained by a microplate reader.

For live/dead staining, 4T1 cells were inoculated into a 6-well cell culture plate, and TTB NPs (30.0 μg mL^-1^) were added to incubate for 4 h after adherence. The 1064 nm laser (1.0 W cm^-2^, 10 min) was used to irradiate the cells of PBS + L and NPs + L groups. Four hours later, Calcein AM (2 μM) and PI (10 μM) were added for incubation for 30 min. Then the wells were washed with PBS for 3 times and replacing the culture medium without FBS, the cells were observed on a Nikon CLSM (A1R HD25).

**S1.5 Tumor-Xenograft Model**

The Balb/c nude mice (5-7 weeks) were raised at 25 °C and 55–60% humidity atmosphere with a 12 h light/dark cycle consistent conditions. All animal studies were in accordance with the Animal Ethics Committee of Soochow University. The mice were subcutaneously administrated with 4T1 cell suspension (∼2 × 10^6^) into the right forelimb of the mice. When the tumor volume reached ∼80 mm^3^, these tumor-xenograft mice were used to follow experiments.

**S1.6 *In Vivo* PA Imaging**

TTB-2 NPs (0.5 mg mL^-1^, 100 μL) were injected into randomly selected 4T1 tumor-xenograft nude mice through the tail vein, and PA imaging was observed using a Vevo LAZR Imaging System in the PA mode at different dosing times.

**S1.7 *In Vivo* Photothermal Therapy**

The 20 nude mice with a tumor volume of 80±10 mm^3^ were randomly divided into 4 groups (n=5): PBS, PBS + L, TTB-2 NPs, and TTB-2 NPs + L. PBS (100 μL) or TTB-2 NPs (1.0 mg mL^-1^, 100 μL) were injected into mice respectively. After 9 hours, the mice tumor of PBS + L and TTB-2 NPs + L group was irradiation by 1064 nm laser (1.0 W cm^-2^, 5 min). After that, mice's weight change and tumor volume change were recorded every 2 days, and the volume calculation formula was as follows: V=long*weith^2^/2. At 14 days, collected the blood of mice was for a routine blood test and blood biochemical test, and euthanized mice and peel off tumors for H&E staining and TUNEL staining. And other organs including heart, liver, spleen, lung, and kidney were euthanized for H&E staining to evolute biosafety.

**S1.8 *In Vivo* Photothermal Therapy Combined with Immunotherapy**

The 4T1 tumor-bearing Balb/c mice with a tumor volume of 80±10 mm^3^ were randomly divided into 6 groups (n=5) on day 7: PBS, PBS+L, TTB-2 NPs, and TTB-2 NPs + L, αPD-1 and TTB-2 NPs + L + αPD-1 group. Then different photothermal therapy were given respectively. The αPD-1 and TTB-2 NPs + L + αPD-1 group were given αPD-1 solution (1 mg mL^-1^, 100 μL) every 3 days from the 8th day to the end of treatment. And different group mice were all given 4T1 cell suspension (5×10^6^) through intravenous injection on the 9th day. The tumor and lung tissue of mice were collected for subsequent analysis after 18 days.

**S1.9 *In Vivo* Flow Cytometry for T Cells and DCs Analysis**

The mice were sacrificed on day 18, and the tumors were peeled off and digested with 0.2% collagenase II at 37 ℃ for 30 min. The cell suspension was filtered through a 70 μm cell strainer. The viable cells were stained with LIVE/DEAD^TM^ Fixable Dead Cell Stain Kit (1:200) at 4 ℃ for 20 min. Then, the cells were blocked with mouse serum for 10 min and stained with anti-mouse CD3-FITC (1:100), anti-mouse CD4-PE/Cyanine7 (1:100), anti-mouse CD8a-APC/Cyanine7 (1:100), anti-mouse CD80-PE (1:100) and anti-mouse CD86-APC (1:100) antibodies at 4 ℃ for 30 min. The stained cells were washed 2 times and resuspended in 200 μL staining buffer for flow cytometric analysis.

**S1.10 *In Vivo* ELISA for TNF-α, IFN-γ, and IL-6 assay**

Mouse blood was collected on day 18. Then blood was left for 1 hour and centrifuged for 10 min at 2000 rpm to receive serum. The serum concentrations of TNF-α, IFN-γ, and IL-6 were analyzed with ELISA kits according to the manufacturer’s protocols.

**S1.11 Statistical Analysis**

Data were expressed as means ± S.D. The data were analyzed by two-tailed Student’s *t-tests*. ****P*<0.001, ***P*<0.01 and **P*<0.05 were considered statistically significant. n.s. represents no significance.

**S2 Supplementary Figures**

**
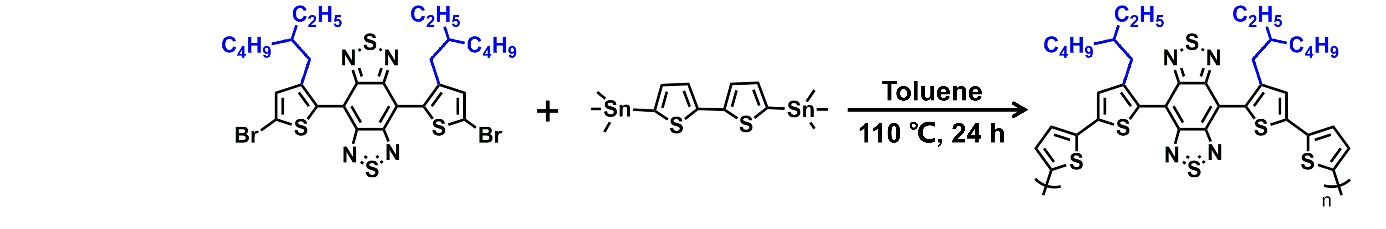
**

**Fig. S1.** Synthetic route of TTB-1.


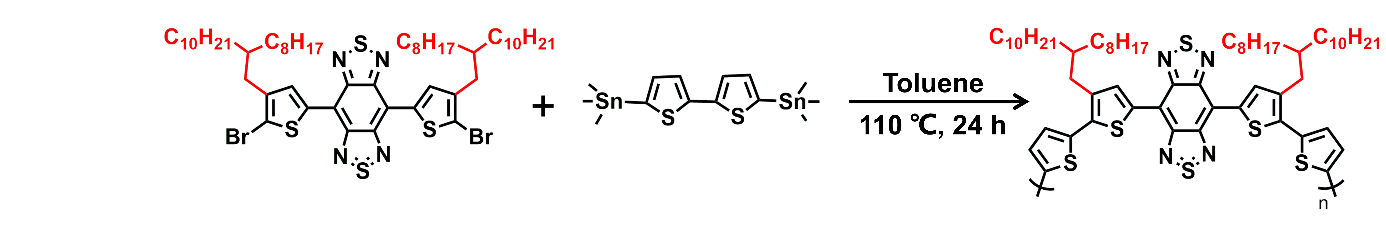


**Fig. S2.** Synthetic route of TTB-2.


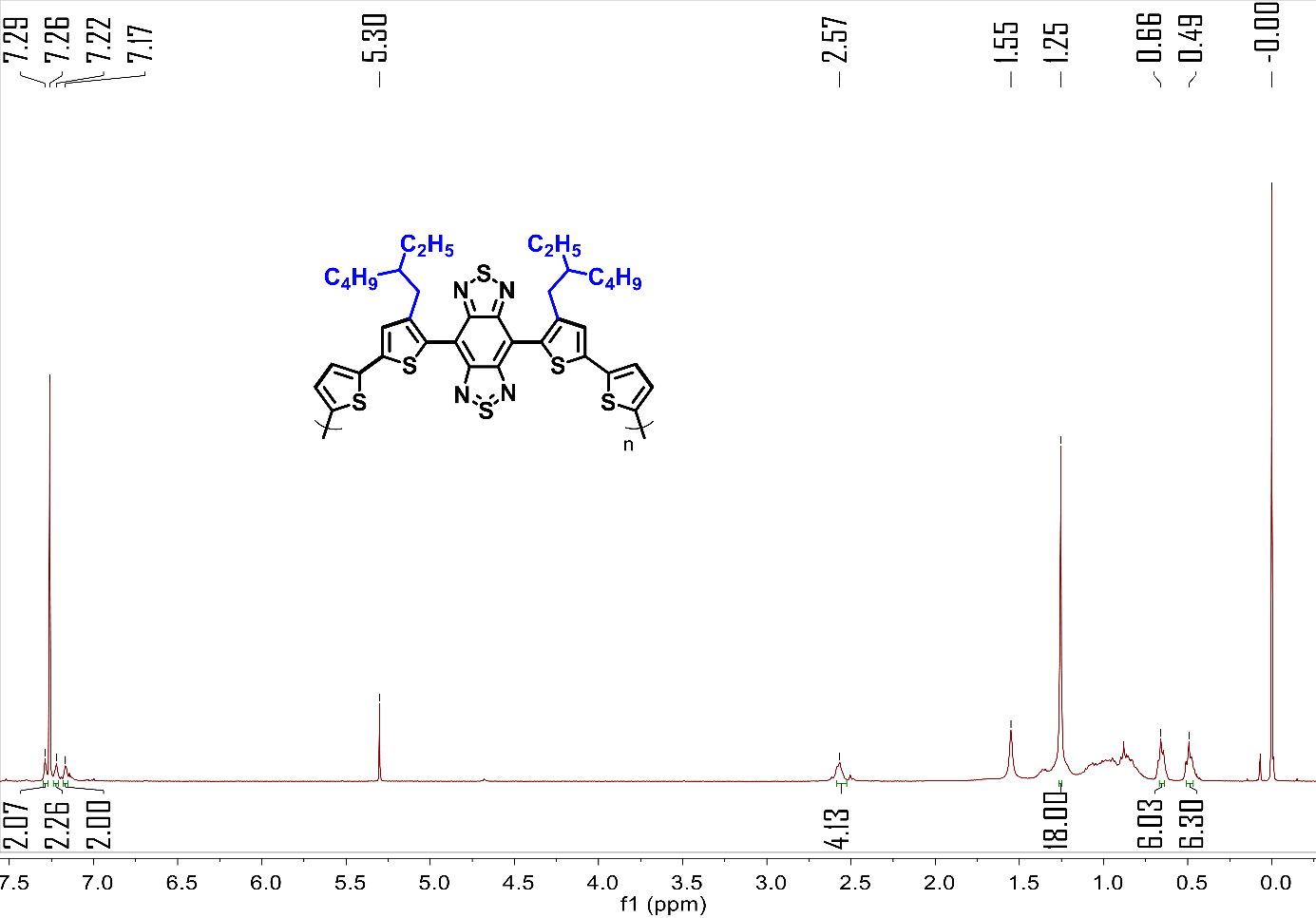


**Fig. S3.** ^1^H NMR (400 MHz, CDCl_3_) of TTB-1.


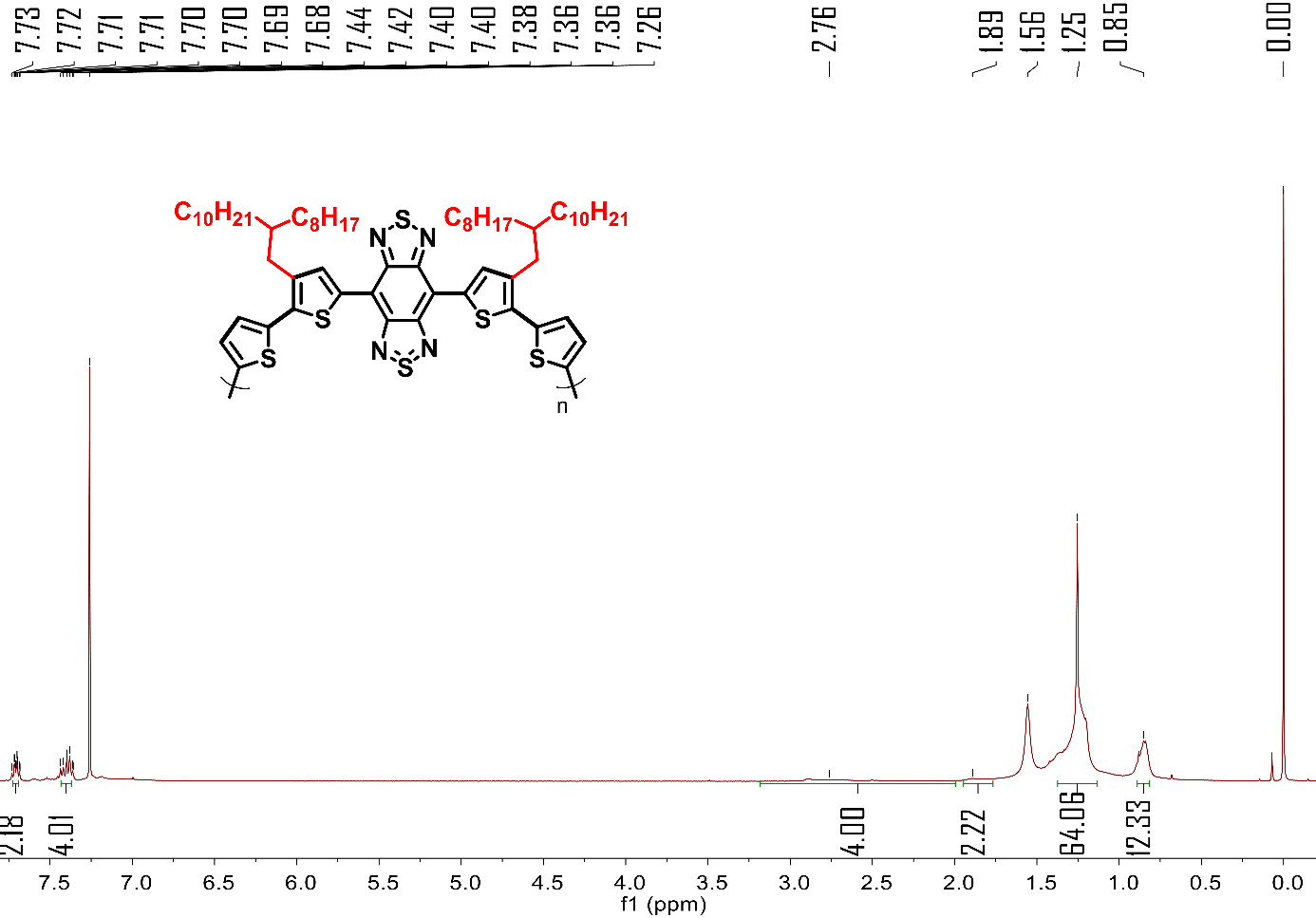


**Fig. S4.** ^1^H NMR (400 MHz, CDCl_3_) of TTB-2.


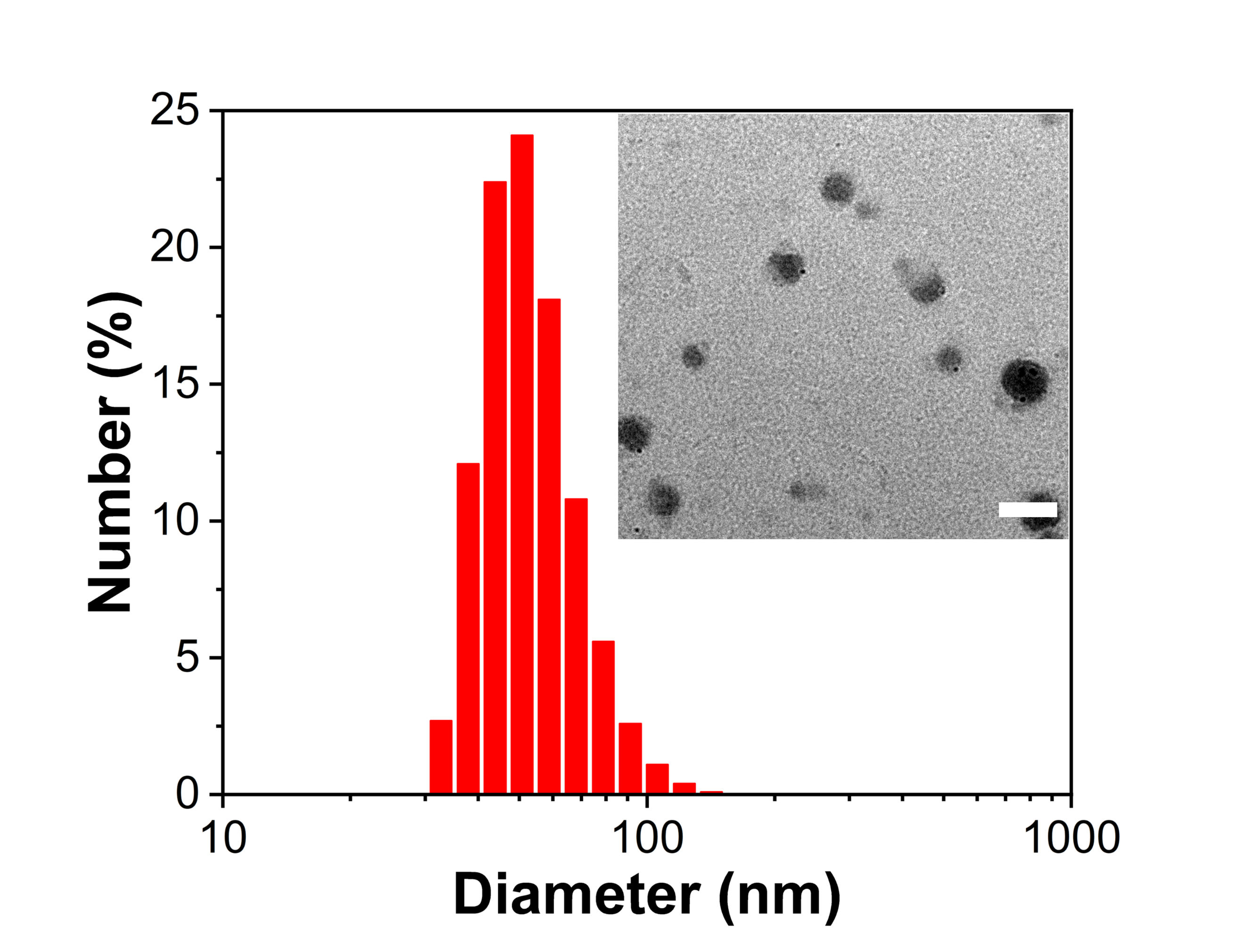


**Fig. S5.** DLS and TEM of TTB-1 NPs. Scale bar: 100 nm.





**Fig. S6.** Heating curves of TTB-2 NPs (50 μg mL^-1^) upon 1064 nm illumination with different laser intensity.





**Fig. S7.** Size of TTB-2 NPs before and after irradiation.

*

*

**Fig. S8.** ESR spectrum of TTB-2 NPs before and after irradiation.





**Fig. S9.** Size change of TTB-2 NPs after 28 days of storage.

*

*

**Fig. S10.** ESR spectrum of TTB-2 NPs after 7 days of storage.


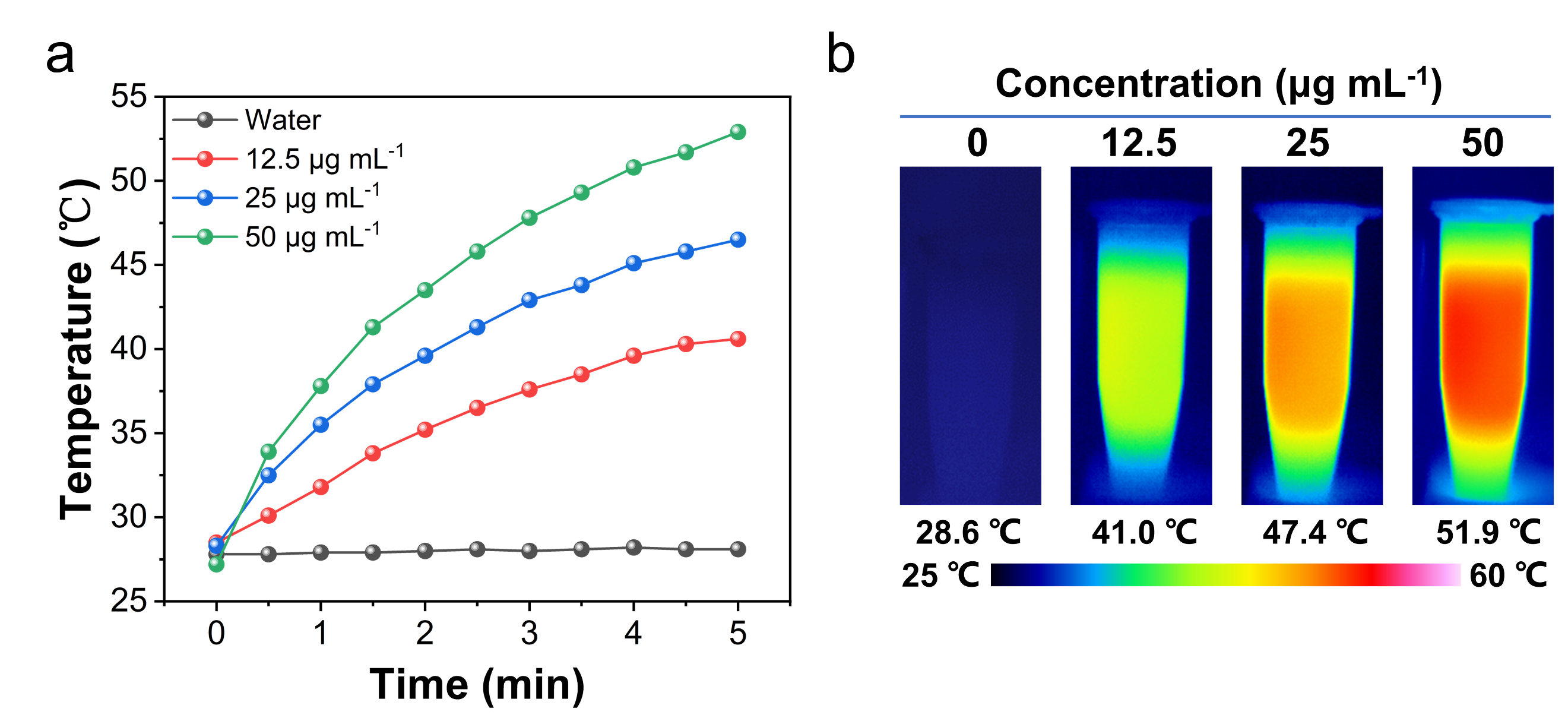


**Fig. S11.** a) Heating curves and b) infrared thermal imaging of TTB-1 NPs upon 808 nm (1.0 W cm^-2^,) illumination with different concentrations.





**Fig. S12.** Heating curves of TTB-1 NPs (50 μg mL^-1^) upon 808 nm illumination with different laser intensities.





**Fig. S13.** Temperature change of TTB-1 NPs aqueous solution after 10 min illumination of 808 nm laser (1.0 W cm^-2^,) and then cooling, with a plot of time against the negative natural logarithm of temperature change during the cooling process.





**Fig. S14.** Photothermal stability of TTB-1 NPs upon five on/off cycles of 808 nm laser illumination (50 μg mL^-1^, 1.0 W cm^-2^).


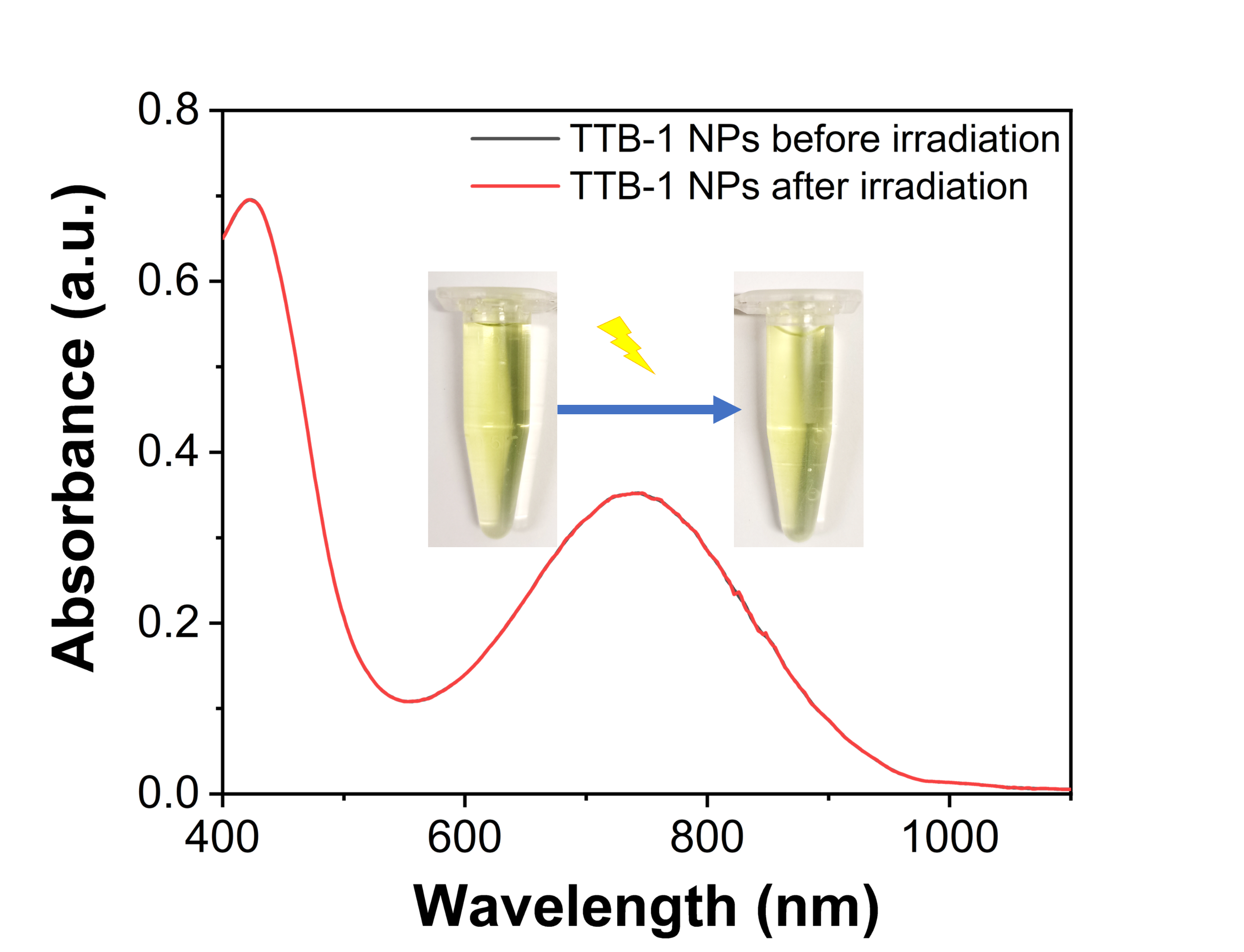


**Fig. S15.** UV-Vis-NIR absorption spectra and photographs of TTB-1 NPs before and after irradiation.


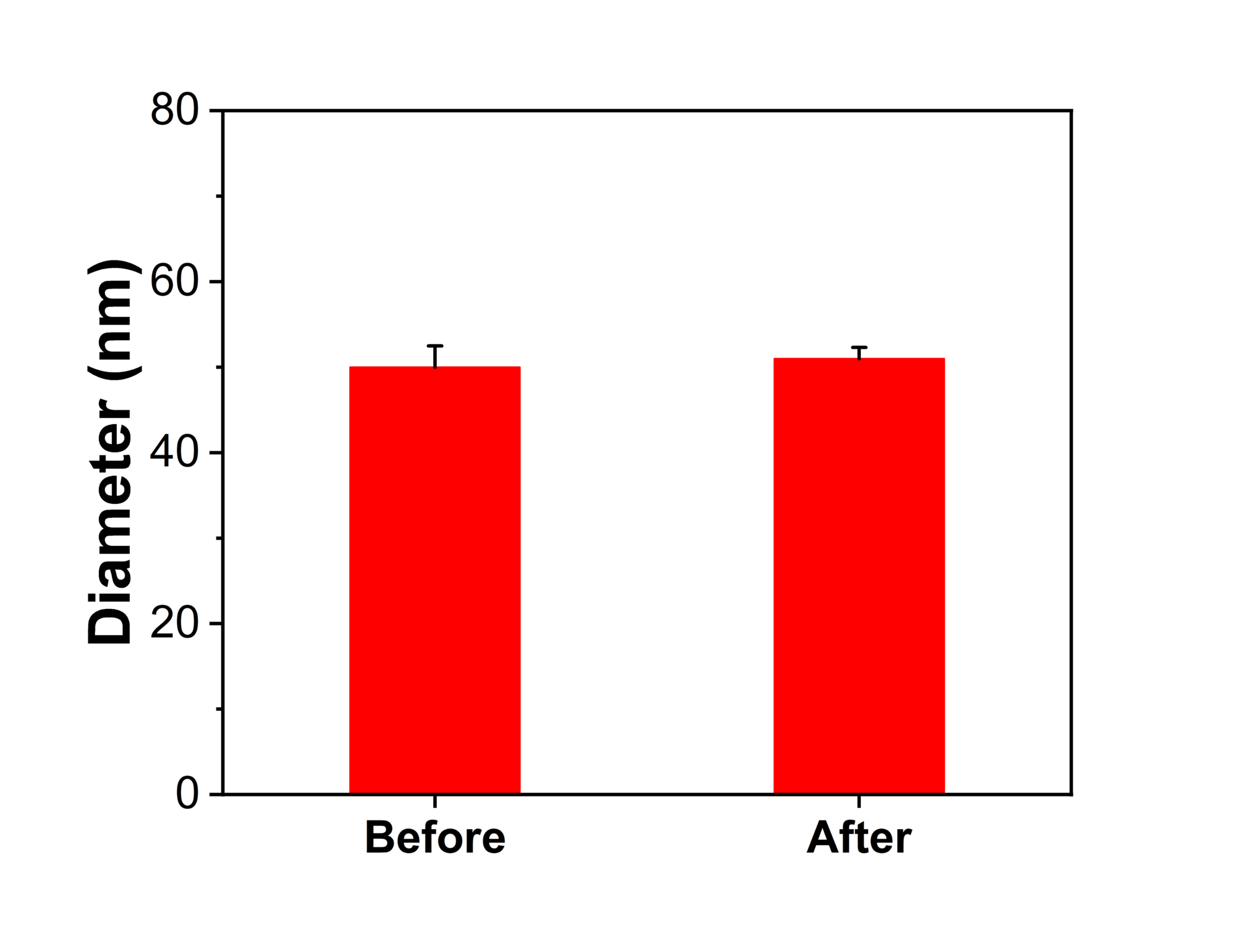


**Fig. S16.** Size of TTB-1 before and after irradiation.


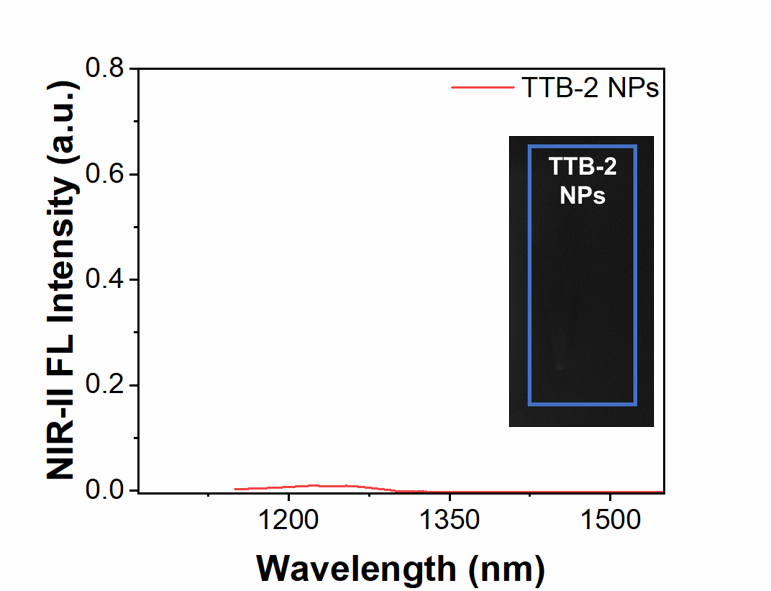


**Fig. S17.** The fluorescence spectrum and NIR-II fluorescence imaging of TTB-2 NPs.


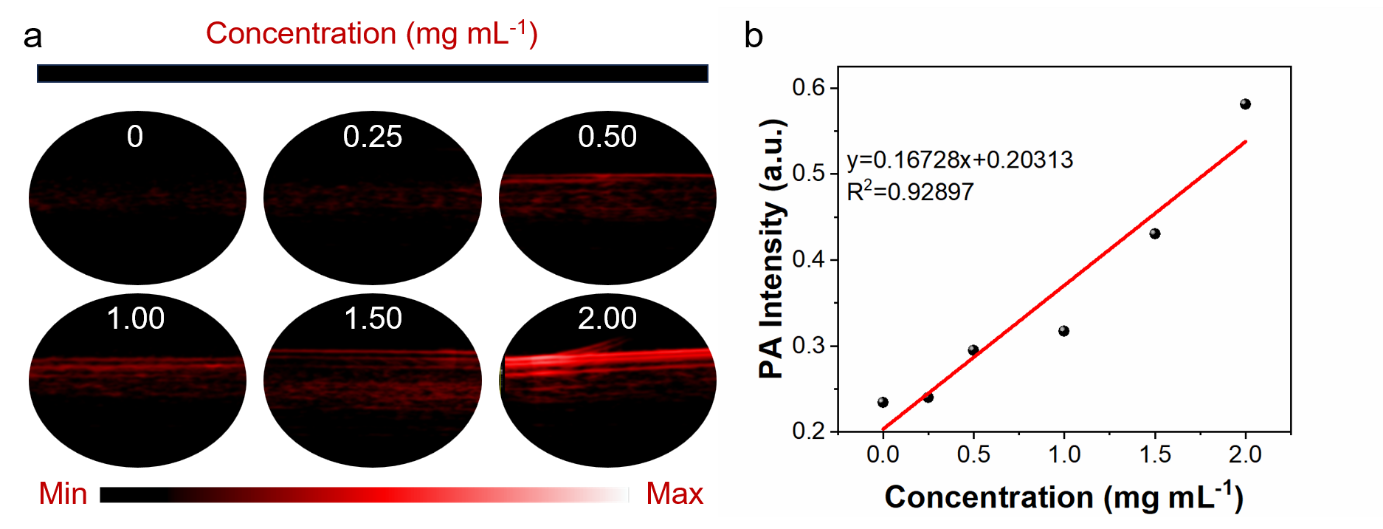


**Fig. S18.** The photoacoustic imaging and corresponding intensity of TTB-2 NPs at different concentrations.


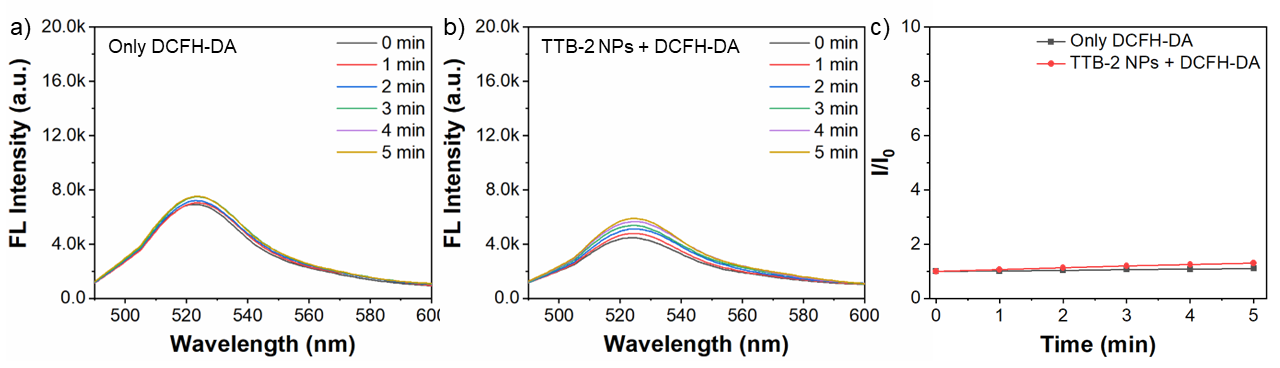


**Fig. S19.** The ROS generation ability of TTB-2 NPs upon the 1064 nm laser irradiation by DCFH-DA.


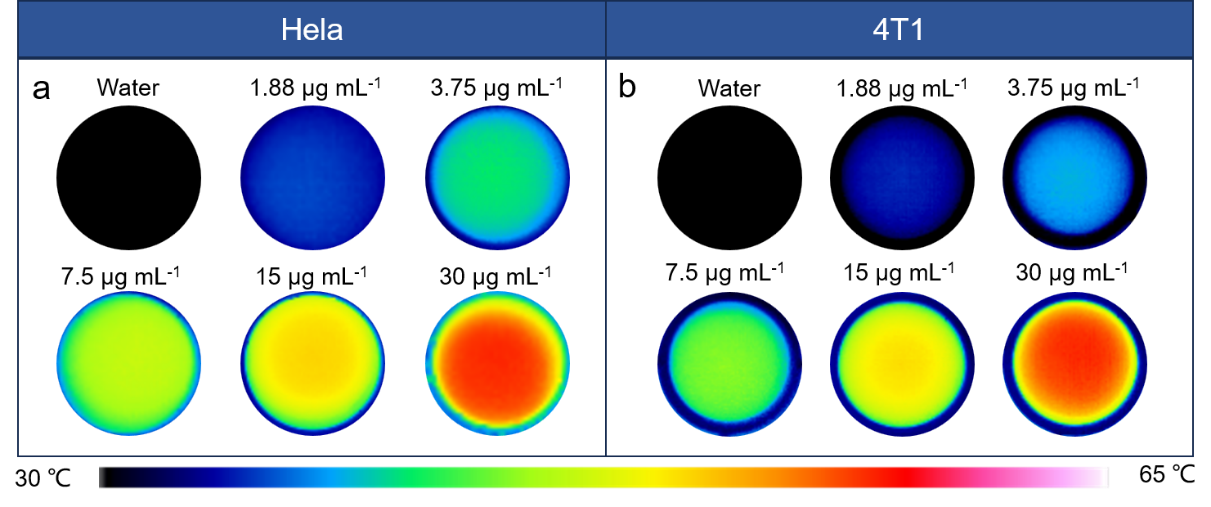


**Fig. S20.** The IR imaging of temperature in MTT assay for a) HeLa and b) 4T1 cells.


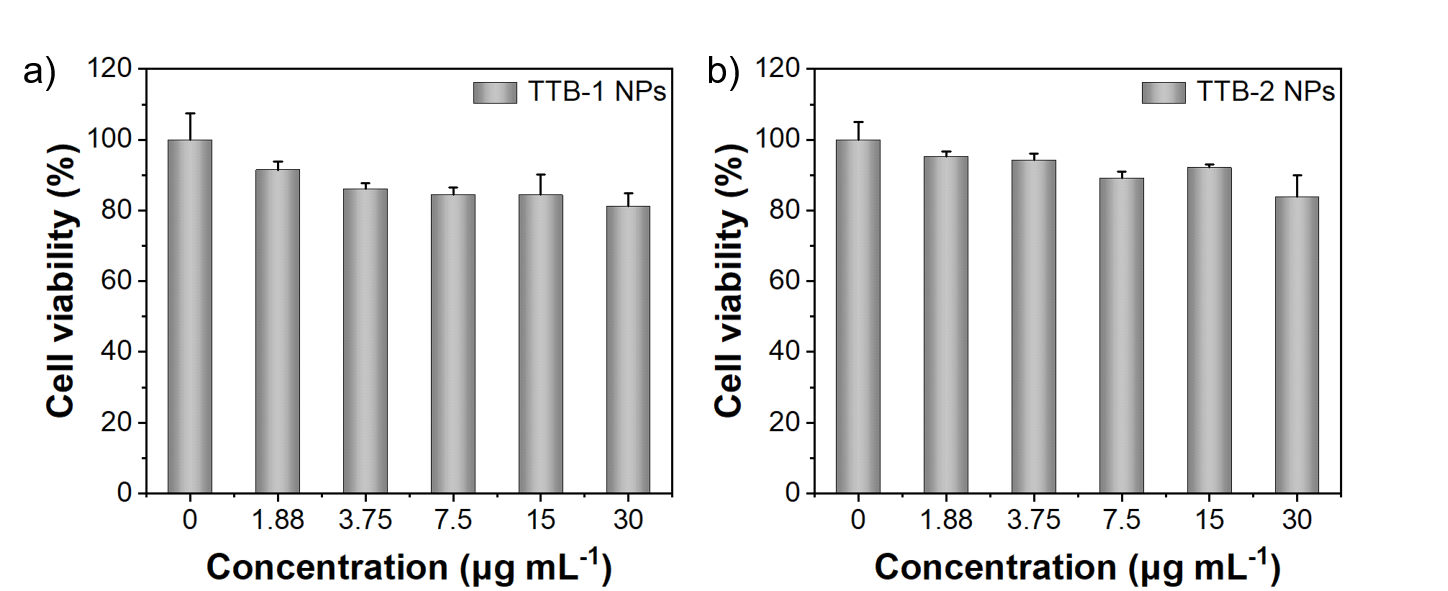


**Fig. S21.** Cell viability of L929 cells after treated with TTB-2 NPs at different concentrations.





**Fig. S22.** The quantitative analysis of tumor photoacoustic signals at different times of TTB-2 NPs post-injection.


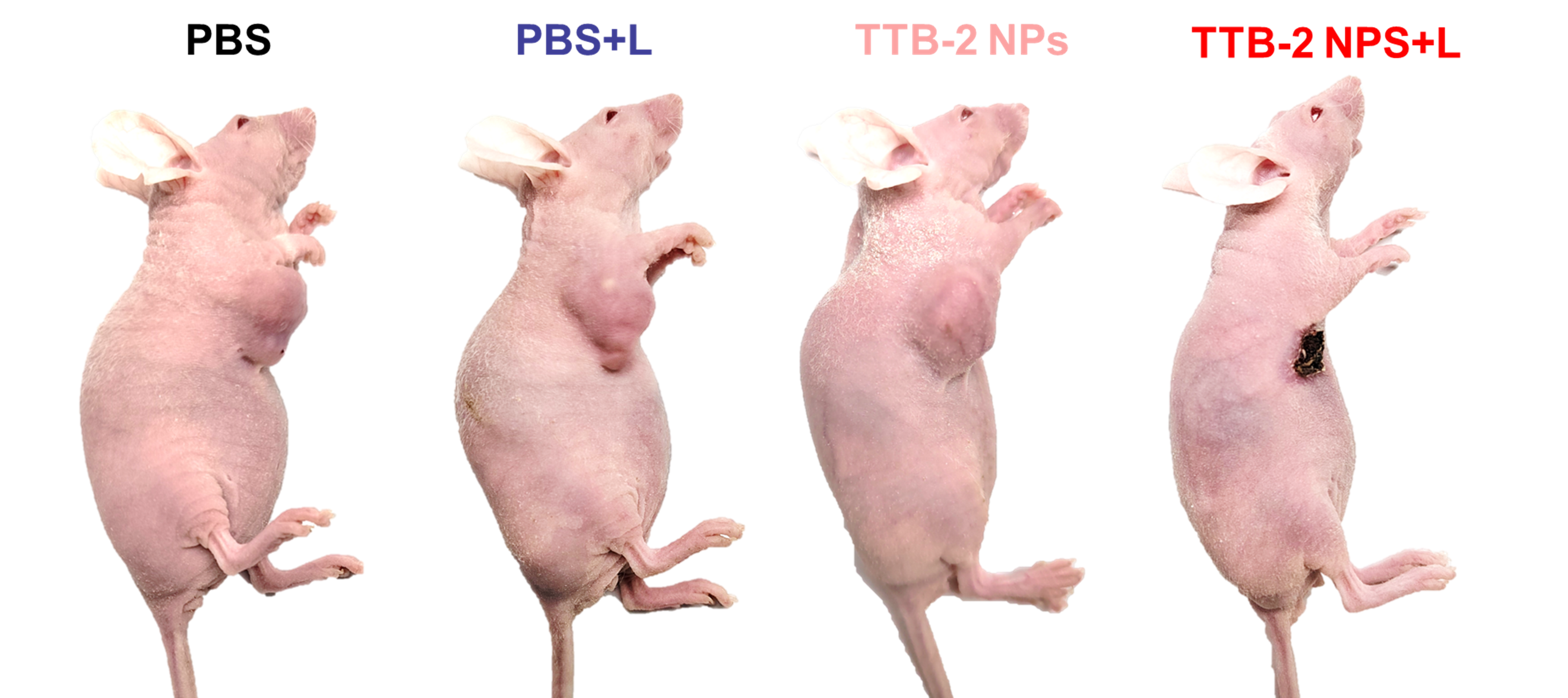


**Fig. S23.** Photograph of mice with different treatments after 14 days.





**Fig. S24.** Body weight of mice with different treatments in 14 days.


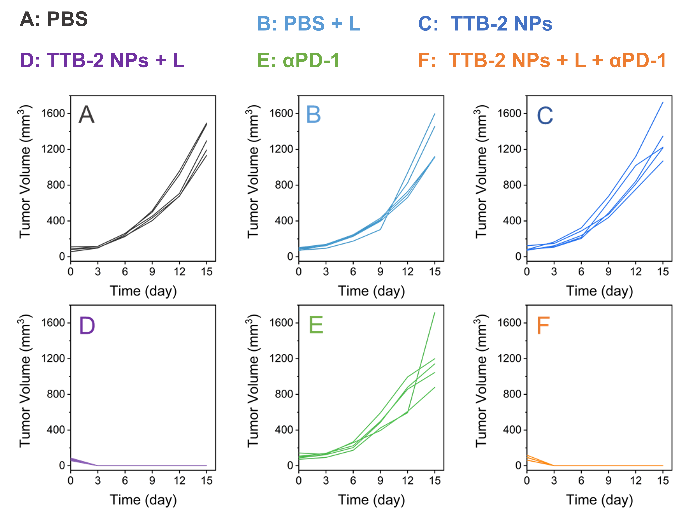


**Fig. S25.** Tumor growth curves of 4T1 tumor-bearing mice under different treatments in lung metastasis model.


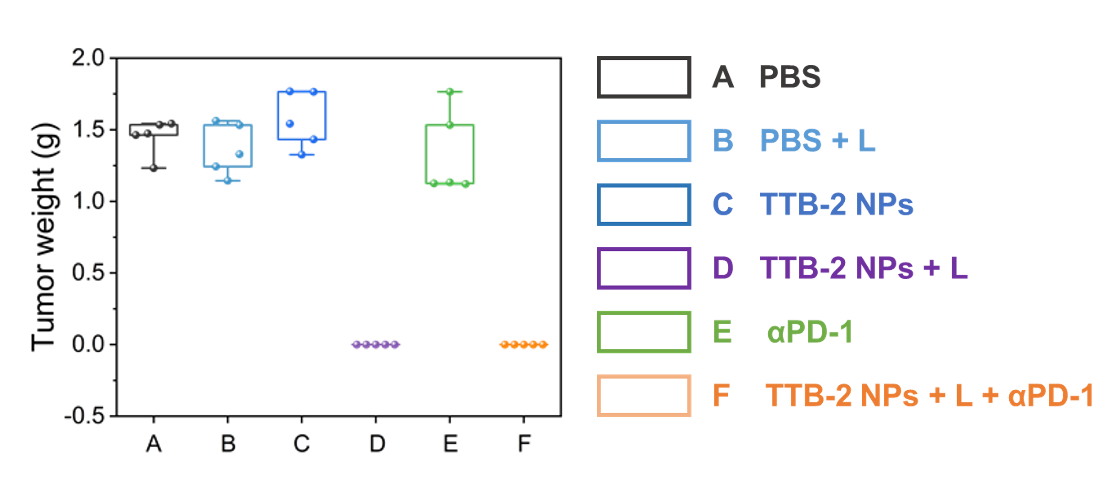


**Fig. S26.** Tumor weight of 4T1 tumor-bearing mice under different treatments in lung metastasis model.





**Fig. S27.** Body weight of 4T1 tumor-bearing mice under different treatments in lung metastasis model.

**
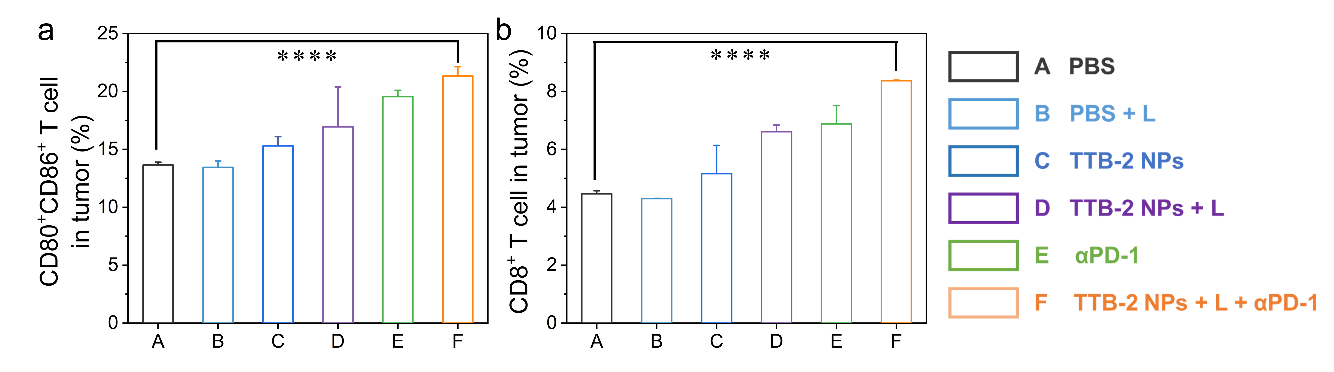
**

**Fig. S28.** a) Quantitative analysis of CD80^+^ CD86^+^ T cells and in tumors from 4T1 tumor-bearing mice after different treatments. b) Quantitative analysis of CD8^+^ T cells (CD3^+^CD8^+^) in tumors from 4T1 tumor-bearing mice after different treatments. **** *P*<0.0001, n=3.

**Table S1.** Summary of photothermal agents under 1064 nm laser irradiation.

| Categories | Agents | PCE (%) | | Ref. |
| --- | --- | --- | --- | --- |
| Organic PTAs | TTB-2 NPs | | 87.7 | This work |
|  | BAF4 | | 80 | *Angew. Chem. Int. Ed. 2021, 60, 22376.* |
|  | IR-SS | | 77 | *Adv. Mater. 2020, 32, 2001046.* |
|  | O-T | | 73 | *Small 2023, 19, 2205640.* |
|  | DTPRR9 | | 70.4 | *Nano Today 2021, 41, 101312.* |
|  | PBTPBF-BT | | 66.4 | *Biomaterials 2018, 155, 103.* |
|  | BDT-TBZ CPNs | | 55 | *Nanoscale 2021,13, 13410.* |
|  | pPCP-NPs | | 51.5 | *Adv. Sci. 2022, 9, 2200732.* |
|  | PTAx | | 47 | *Adv. Mater. 2022，34, 2105976.* |
|  | TTQ-MnCO | | 44.43 | *Biomaterials 2022, 280, 121319.* |
| Inorganic PTAs | Ni-CDs | | 76.1 | *Adv. Funct. Mater. 2021, 31, 2100549.* |
|  | PDMO1064 | | 69.15 | *J. Mater. Chem. B 2021, 9, 6441.* |
|  | CSC-2@PEG | | 51.5 | *Carbon 2022, 197, 98.* |
|  | MoO_3_-x | | 46.99 | *small 2021, 17, 2007486.* |
|  | PtAg | | 45.7 | *Adv. Sci. 2021, 8, 2100386.* |
|  | CFPP NPs | | 43.2 | *Anal. Chem. 2022, 94, 5317.* |
|  | Bi_19_S_27_I_3_ | | 41.5 | *Nanoscale 2021, 13, 5369.* |

**References**

1. L. S. Li, Q. Deng, Y. Zhang, X. Li, G. Wen, et al., Rational design of conjugated small molecules for superior photothermal theranostics in the NIR-II biowindow. Adv. Mater. **32**(33), e2001146 (2020). <https://doi.org/10.1002/adma.202001146>
